# *Hilnc-*mediated UCP1 translation repression contributes to thermogenesis and energy expenditure

**DOI:** 10.7150/thno.122688

**Published:** 2026-01-01

**Authors:** Man Jiang, Yu Li, Yiao Jiang, Runze Wang, Jiayin Peng, Yuang Wang, Zhen Qu, Yi Chang, Zhao Zhang, Yun Zhao

**Affiliations:** 1 Key Laboratory of Multi-Cell Systems, Shanghai Institute of Biochemistry and Cell Biology, Center for Excellence in Molecular Cell Science, Chinese Academy of Sciences, University of Chinese Academy of Sciences, Shanghai, China.; 2 School of Life Science and Technology, ShanghaiTech University, Shanghai, China.; 3 Center for the Genetics of Host Defense, University of Texas Southwestern Medical Center, Dallas, TX, USA.; 4 Division of Endocrinology, Department of Internal Medicine, University of Texas Southwestern Medical Center, Dallas, TX, USA.; 5 Department of Medical Aesthetic, Yangpu Hospital, Tongji University School of Medicine, Shanghai, China.; 6 School of Life Science, Hangzhou Institute for Advanced Study, University of Chinese Academy of Sciences, Hangzhou, China.

**Keywords:** beige adipocytes, UCP1, long non-coding RNA, translational regulation, thermogenesis

## Abstract

**Background**: Beige adipocytes play a critical role in thermoregulation by upregulating uncoupling protein 1 (UCP1) upon stimulation. While the transcriptional regulation of UCP1 in adipose tissue has been extensively investigated, the mechanisms governing its translational control remain largely elusive.

**Methods**: A cold exposure protocol was employed to induce beige adipocyte biogenesis in mouse subcutaneous fat. The overall metabolic rate of mice was monitored by metabolic cage. Primary adipocyte precursors were isolated from the stromal vascular fraction (SVF) of inguinal white adipose tissue (iWAT) and differentiated into beige adipocytes using a standard adipogenic induction cocktail. Transmission electron microscopy (TEM) was utilized to examine mitochondrial morphology. Functional rescue experiments were performed via adenovirus-mediated gene overexpression. Potential binding partners were screened by LC-MS/MS, while RNA immunoprecipitation (RIP) and RNase protection assay (RPA) were applied to evaluate RNA-protein and RNA-RNA interactions, respectively. Additional mechanistic insights were obtained through qPCR, Western blotting, Immunohistochemistry and bioinformatics analyses.

**Results**: In this study, we discovered that *Hilnc*, a long non-coding RNA (lncRNA), functions in beige adipocytes by suppressing UCP1 translation. Adipocyte-specific *Hilnc*-deficient mice display increased energy expenditure, elevated body temperature, smaller inguinal white adipose tissue volume and coupling efficiency, and elevated UCP1 protein level. *Hilnc* binds to the 3' untranslated region of *Ucp1* mRNA and recruits insulin-like growth factor 2 binding protein 2 for translational suppression. The previously characterized human *Hilnc* functional homolog negatively correlates with UCP1 protein levels in human adipose tissues and suppresses *UCP1* translation via similar mechanisms.

**Conclusion**: Our findings highlight *Hilnc*'s post-transcriptional role in thermoregulation in beige adipocytes and offer new insights into the variability of thermogenesis among individuals.

## Introduction

Adipose tissues are recognized as important players in the maintenance of energy homeostasis of various organisms, with recent studies highlighting their diverse roles including body temperature regulation, hormone production, and immune response modulation 1-5. Beige adipocytes represent a lineage of adipocytes that undergo reprogramming in response to external stimuli such as low temperatures and hormones, and express thermogenic genes similar to those observed in brown adipose tissues (BATs), thereby acquiring thermogenic capabilities 6-8. The thermogenic abilities of BATs and beige adipocytes are largely attributed to their cellular mitochondria 9. Brown and beige adipocytes contain more mitochondria than their white adipocyte counterparts, and these mitochondria have a higher concentration of the uncoupling protein UCP1 in their inner membranes. UCP1 functions as a thermogenic protein by uncoupling the mitochondrial electron transport chain from ATP production, allowing protons to flow freely into the mitochondrial matrix, thereby dissipating energy as heat. UCP1 is central to non-shivering thermogenesis in mammals, and its expression level directly correlates with thermogenic activity under varying physiological conditions 10-13. The expression of UCP1 in adipose tissues is regulated through complex mechanisms, with existing research predominantly focusing on transcription factor-mediated regulation 14-25. However, the post-transcriptional regulation of UCP1, including mechanisms controlling mRNA stability and translation, remains less well understood 26-28. A deeper understanding of these post-transcriptional processes is essential to fully elucidate how UCP1 levels are regulated and how thermogenic capacity is controlled.

Long non-coding RNAs (lncRNAs) are a group of RNA molecules longer than 200 nt that possess no protein potential or only contain small open reading frames 29. LncRNAs were found to modulate cellular gene expression at multiple levels, including transcription, translation, and post-translational control 30, 31. In recent years, lncRNAs have been identified as key regulators of metabolic processes and contributors to the development of metabolic disorders. They modulate lipid absorption, metabolism, adipocyte differentiation, and adjust body energy balance and thermogenesis 32-37. Although several lncRNAs have implicated in the transcriptional regulation of UCP1 36, their potential role in post-transcriptional control has remained unexplored and unanticipated.

Our laboratory previously identified *Hilnc*, a Hedgehog signaling pathway-induced lncRNA, and demonstrated its role in regulating lipid metabolism in the liver. The *Hilnc* gene is located on chromosome 10 (chr10: 39,432,067-39,489,678). According to the latest NCBI records, the mature *Hilnc* transcript is 801 nucleotides in length, which includes a polyA tail. Functionally, we observed that both *Hilnc^BM/BM^* mice (carrying a Gli-binding site mutation in the *Hilnc* promoter) and *Hilnc* knockout mice are protected from high-fat diet-induced obesity and the development of hepatic steatosis 34. We subsequently found that *Hilnc* also affects various adipose tissues, promoting further investigation into its potential role in adipocyte biology. In this study, we show that adipocyte-specific *Hilnc-*deficient mice display altered inguinal white adipose tissue (iWAT) morphology and increased overall energy expenditure, which is linked to increased adipocyte beiging in iWATs. Mechanistically, *Hilnc* suppresses UCP1 protein levels in beige adipocytes by inhibiting *Ucp1* mRNA translation. The human homolog of *Hilnc* (*h-Hilnc*), previously discovered by our group, is expressed in human adipose tissues and suppresses *UCP1* mRNA translation via a similar mechanism. These findings reveal a previously unrecognized, evolutionarily functionally conserved role for lncRNAs in regulating thermogenesis through post-transcriptional mechanisms, and highlight the therapeutic potential of targeting lncRNAs to modulate systemic energy homeostasis.

## Materials and Methods

### Human sample acquisition and Ethics Statement

All human adipose tissue samples were obtained from the Yangpu District Central Hospital of Shanghai, China, with sampling conducted after informed consent was obtained. Sex was not considered as a biological variable. The study was approved by the Institutional Review Board of the Center for Excellence in Molecular Cell Science (CEMCS), Chinese Academy of Sciences (approval no. 2024-164).

### Animals

All mice used in this study have a C57BL/6 background. Sex was not considered as a biological variable. The *Hilnc^f/f^* mice were generated using CRISPR-Cas9 technology (provided by Cyagen Biosciences). The loxP sites were inserted upstream of exon 2 and downstream of exon 3 of the *Hilnc* genomic locus. *Hilnc^f/f^* mice were crossed with *AdipoQ-Cre* mice (Jackson Laboratory, 028020) or *UCP1-Cre* mice (a thankful gift from Prof. Hao Ying at Shanghai Institute of Nutrition and Health, Shanghai, China) as indicated in the previous reports 38, 39.

For room temperature experiments, mice were housed in an environment maintained at a temperature of 22-25 °C and a humidity of 40-60%, with a 12/12 h light-dark cycle. To completely eliminate the confounding effects of cold stress, a subset of mice was maintained at a thermoneutral temperature of 30 °C and a humidity of 40-60%, with a 12/12 h light-dark cycle. For 4 °C cold acclimation model, mice were housed for 1-3 weeks in an environmental chamber (Forma™, Thermo Scientific) maintained at temperature of 4 °C and humidity of 40-60%, with a 12/12 h light-dark cycle. For cold re-exposure experiments, mice that have undergone cold acclimation for 3 weeks were returned to room temperature for 1 h, then returned to the cold environmental chamber for 12 h. Rectal temperatures of mice were measured using BAT-12 Microprobe Thermometer with RET-3 Rectal Probe for Mice (Physitemp). Mice were fed either a high-fat diet (HFD; 60% fat; Research Diets) or a normal chow diet (NCD) and had free access to water. Body composition (fat and lean mass) was determined by NMRI (NIUMAG analytical instrument, NM42-060H-I). During experiments, mice were processed between 12:00 and 18:00. All mouse experiments were conducted in accordance with the guidelines of the Institutional Animal Care and Use Committee of the Institute of Biochemistry and Cell Biology, Shanghai Institutes for Biological Sciences, Chinese Academy of Sciences (SIBCB-S313-2305-14).

### Cell Culture and Transfection

3T3-L1, NIH 3T3, and HEK293T cell lines were cultured in high-glucose Dulbecco's Modified Eagle Medium (MeilunBio, MA0212) supplemented with 10% fetal bovine serum (Gibco, A5256701) and 1% penicillin-streptomycin (MeilunBio, MA0110-1). Cells were maintained in a humidified incubator at 37 °C with 5% CO₂ and passaged every 2-3 days at a 1:4 split ratio.

For the cell transfection experiments, the Lipofectamine™ 3000 reagent (Invitrogen, L3000015) was used to transfect the plasmids according to manufacturer's recommendations. Samples were collected 48 h post-transfection for further analysis.

### Plasmids

Fragments required for overexpression was cloned into the pcDNA3.1(+) vector using standard cloning techniques. These fragments including full-length *Hilnc* cDNA, *HilncΔ450-600*, *Ucp1* mRNA, *Igf2bp2* CDS, *UCP1* CDS, *UCP1* CDS + 3'UTR. Empty pcDNA3.1(+) vectors were used as negative controls. pCDH (GFP-tag) were used as a transfection efficiency reference.

The construction of the PsiCHECK-2 plasmid system involved inserting the 3'UTR regions of *Ucp1* mRNA and *UCP1* mRNA downstream of the Renilla luciferase CDS in the PsiCHECK-2 plasmid. This setup was used to assess the impact of the *Ucp1* and *UCP1* 3'UTRs on protein translation efficiency, respectively. Firefly luciferase served as the internal control in this dual-luciferase system.

These sequences were verified by Sanger sequencing.

### Metabolic Phenotyping

Metabolic characteristics of the mice were assessed using a custom indirect calorimetry system (Columbus × Oxymax/CLAMS). Mice were housed individually in metabolic cages for 72 h to measure oxygen consumption (VO₂), carbon dioxide production (VCO₂), respiratory exchange ratio (RER), energy expenditure (EE), locomotor activity, and food intake.

For the Norepinephrine Bitartrate Monohydrate (NA) injection experiment, 2 mg/kg NA (MCE, HY-13715B) dissolved in PBS was administered via intraperitoneal injection to the mice. Metabolic parameters were monitored for 1 h post-injection. The mice resumed normal activities within 1 h after NA injection and showed no adverse effects.

Data were collected and analyzed using the Oxymax software.

### Histology, Immunohistochemistry & Oil red O stain

Freshly dissected mouse brown adipose tissues (BATs), epidydimal white adipose tissues (eWATs), and inguinal white adipose tissues (iWATs) (all from 8 weeks old mice) were fixed in 4% paraformaldehyde and embedded in paraffin. Paraffin-embedded sections (4μm) were stained using Histostain™-SP Kit (ZSGB-bio, SPN-9002), with the specific steps carried out according to the instructions provided in the kit. Briefly, for permeabilization before staining, sections were treated with PBS containing 0.1% Triton X-100. Antigen retrieval was performed using sodium citrate antigen retrieval solution (Solarbio, C1032-100). The retrieval process involved immersing the sections in the antigen retrieval solution and heating them in a pressure cooker to full pressure, followed by continued heating for 10 min. The sections were allowed to cool to room temperature, then stained with anti-UCP1 (Abcam, ab10983-50µL) or anti-TOM20 (Proteintech, 11802-1-AP) antibodies, respectively. The hematoxylin and eosin (H&E) staining was performed according to the standard protocol (Beyotime, C0105M).

For Oil red O stains to assess lipid content within cells, differentiated cells were washed once with PBS. Cells were fixed with 10% formaldehyde solution for 30 min at room temperature. After two washes with distilled water, cells were permeabilized with 60% isopropanol for 5 min. Freshly prepared Oil Red O (Sigma, O0625) working solution was added to cover the cells and incubated on a rocker at room temperature for 10-20 min. Cells were gently washed with distilled water to remove excess stain. Lipid droplets were visualized under a microscope as red droplets. Quantification of lipid droplet size from Oil Red O-stained images was performed using ImageJ.

### Western Blotting

Cells were lysed in cell lysis buffer (Cell Signaling Technology, 9803S) supplemented with protease inhibitor cocktail (Targetmol, C0001). The lysates were sonicated and centrifuged until the cell lysate was clear. Tissues were homogenized in ice-cold cell lysis buffer containing protease inhibitor cocktail with tissue homogenizer (Bead Ruptor 24 Elite, OMNI International). The tissue or cell lysates were resolved on 10% SDS-PAGE gels and then electrotransferred onto PVDF membranes. Proteins were stained with anti-ACTIN (ABclonal, AC038), anti-GFP (Proteintech, 66002-1-IG), anti-UCP1 (Abcam, ab10983-50μL), anti-TOM20 (Proteintech, 11802-1-AP), or anti-IGF2BP2 (Proteintech, 82757-2-RR) overnight at 4 °C, then with HRP-conjugated antibodies of the corresponding species specificities, respectively (anti-rabbit: Invitrogen, 31460; anti-mouse: Invitrogen, 31430). Stained membranes were visualized using Enhanced ECL Chemiluminescent Detection Kit (Vazyme, E411-05) with Tanon 5200 Chemiluminescence Imaging System. Band intensity was quantified using ImageJ.

### Tissue Respiration by Seahorse Analyzer

iWAT blocks (2 mg - 5 mg) were immobilized at the bottom of XF24 Islet Capture Microplates (Agilent Seahorse, 101122-100). Each well was filled with 525 μL of XF DMEM Base Medium (pH 7.4; Agilent Seahorse, 103575-100). During the measurement, the following inhibitors were dissolved in XF DMEM Base Medium and injected sequentially: 75 μL Oligomycin A (final concentration = 15μM) (Selleck, S1478); 75μL of FCCP (final concentration = 20μM) (Sigma, C2920); 75μL of Rotenone (final concentration = 10 μM) (Sigma, R8875-1G) and Antimycin A (final concentration = 10μM) (BioVision, 2247-50MG). Experimental parameters were set up according to the Agilent Seahorse XFe24 Analyzer user manual. GraphPad Prism was used to generate OCR time course plots and bar charts to illustrate the differences between different treatment groups.

### Mitochondrial Isolation and ATP Assay

Mouse iWATs were dissected to remove lymph nodes, and mitochondria were isolated and purified using a mitochondrial isolation kit (Proteintech, PK10016). The purified mitochondria were counted under a microscope with Janus Green B staining. All mitochondrial samples were adjusted to the same uniform density. Subsequently, an equal number of mitochondria from tissues of different genotypes were boiled for 2 min to release ATP, which was immediately measured following the manufacturer's instructions of the ATP assay kit (HY-K0314, MCE) 40. The ATP concentration in the mitochondria was calculated based on the standard curve generated by the kit.

### Stromal Vascular Fraction (SVF) Isolation and Differentiation

SVF isolation was performed according to previous protocols with minor modifications 41, 42. Briefly, iWATs were carefully dissected using ophthalmic scissors and forceps, and lymph nodes were removed. The adipose tissues were placed in sterile Eppendorf tubes and cut into small pieces. The tissues were then transferred to 15 mL centrifuge tubes and digested with approximately 5 times tissue volume of Krebs-Ringer bicarbonate (KRB) buffer (118 mM NaCl, 5 mM KCl, 2.5 mM CaCl_2_, 2 mM KH_2_PO_4_, 2mM MgSO_4_, 25 mM NaHCO_3_, 5 mM glucose, pH 7.4. With 1.5 μg/mL collagenase I (Thermofisher-Invitrogen, 17018029) and 3% BSA) at 37 °C with shaking at 140 rpm for 40 min to help break down the extracellular matrix. After terminating the digestion by adding 1/3 total volume of FBS, the suspensions were centrifuged at 500 × g to remove the upper layer of floating adipocytes and the supernatant. The pellets were washed three times with PBS, and the pelleted SVFs was filtered through a 70 μm cell strainer to obtain single-cell suspensions. The cells were then resuspended in complete culture medium (DMEM/F12 with 14% FBS, 1% Penicillin-Streptomycin, 1% GlutaMAX (Gibco)) and cultured. When the cells reached 120% confluence, they were treated with induction differentiation medium (0.5 mM 3-Isobutyl-1-methylxanthine (IBMX) (Sigma, I5879), 1 μM dexamethasone (Sigma, D4902), 1 μg/mL insulin (Solarbio, I8040), 1 μM rosiglitazone (Targetmol, T0334), 1 nM 3,3',5-Triiodo-l-thyronine (T3) (Sigma, T2877) in complete culture medium) for 2 days. Subsequently, the medium was replaced with maintenance medium (1 μg/mL Insulin and 1 nM T3 in complete culture medium) for 4-6 days until visible lipid droplet accumulation was observed within the cells.

For evaluation of UCP1 protein stability in cells, 50μM cycloheximide (CHX in DMSO solution, MCE, HY-12320) was added to the culture medium after SVF differentiation was completed. Samples were collected at 0 h, 3 h, 6 h, 9 h, 12 h, and 15 h post-treatment for protein lysis and subsequent western blotting analysis.

### Flow Cytometry Analysis of SVF Cells

The digested iWATs were filtered through a cell strainer, washed with FACS buffer (PBS + 2% heat-inactivated FBS), and centrifuged at 500 × g for 10 min at 4 °C. The supernatant was removed, and the pelleted SVF was resuspended in 1mL red blood cell lysis buffer (containing sodium bicarbonate, ammonium chloride, and 0.5 M EDTA; pH 7.4) and incubated at room temperature for 1 min. 10mL cold PBS were added to stop the lysis, and the cells were then filtered through a 70 μm sterile cell strainer and centrifuged at 500 × g for 10 min at 4 °C.

Single cells were stained with anti-CD45 (Biolegend, S18009F, 157203), anti-CD31 (Biolegend, W18222B, 160211), anti-SCA1 (Biolegend, D7, 108107) in FACS buffer for 30 min, followed by viability staining with 1 μg/mL DAPI in FACS buffer for 5 min. Cells were washed twice with PBS and resuspended in PBS for analysis.

### RNA-Seq and Analysis

The workflow comprised the following steps: library preparation & sequencing: total RNA was extracted, and mRNA was enriched using poly-T magnetic beads. After fragmentation and cDNA synthesis, libraries were constructed and sequenced on an Illumina platform following quality control (Bioanalyzer 2100, Qubit, real-time PCR). Data processing: raw reads were processed with fastp for quality control and adapter removal. Clean reads were aligned to the reference genome using Hisat2 v2.0.5. Expression quantification: gene counts were obtained with FeatureCounts v1.5.0-p3 and FPKM values were calculated. Differential analysis: DESeq2 was used to identify differentially expressed genes (threshold: adjusted P-value ≤ 0.05 and |log2(foldchange)| ≥ 0.5). Pathway analysis: KEGG pathway enrichment was tested using clusterProfiler.

### RNA Extraction, Reverse Transcription-Polymerase Chain Reaction (RT-PCR) and Quantitative PCR (RT-qPCR)

Total RNA was extracted from cells by directly adding Trizol (Invitrogen) and homogenizing, and from tissues by adding Trizol and using a tissue homogenizer to assist in tissue lysis. RNA extraction was performed using the standard method involving chloroform extraction and isopropanol precipitation. For reverse transcription of RNAs into cDNAs, 0.5-1μg of RNA was reverse-transcribed using HiScript III RT SuperMix for qPCR (+ gDNA wiper) (Vazyme, R312-02). For PCR of reverse transcribed products, the reactions were set up with 2 × Phanta Max Master Mix (Vazyme, P515) with the cDNA as template, and the product was resolved on agarose gels. Quantitative PCR (qPCR) was performed using AceQ Universal SYBR qPCR Master Mix (Vazyme, Q511-02) with cDNA as template. Quantification was performed using the 2 ^ (-ΔΔCt) method. Primers used are listed in the Table S4.

### Transmission Electron Microscopy (TEM) Imaging

TEM samples of iWATs were prepared, sectioned, and stained according to the standard protocols provided by the Electron Microscopy Platform of the Institute of Biochemistry and Cell Biology, Shanghai Institutes for Biological Sciences, Chinese Academy of Sciences. The following is a brief description of the sample preparation method: On the first day, excise samples to a size of approximately 1 mm³ and place them in Eppendorf tubes containing 2.5% glutaraldehyde, fully immersing them, and fix overnight at 4 °C. On the second day, aspirate the fixative solution and wash the samples three times with PBS by gentle shaking for 10 min each time. After washing with PBS, rinse the samples with deionized water by gentle shaking. Fix the samples with 1% osmium tetroxide at room temperature for 1.5 h. Aspirate the osmium tetroxide and wash the samples three times with deionized water, each for 10 min. Dehydrate the samples gradually using ethanol solutions of increasing concentrations (30%, 50%, 70%, 80%, 95%, and 100%) for 10 min each, with gentle shaking. Repeat the 100% ethanol dehydration step once. Replace ethanol with 100% acetone twice, each for 15 min with gentle shaking. Infiltrate the samples in a 1:1 mixture of Epon812 resin and acetone by gentle shaking for 2 h, then infiltrate overnight in pure Epon812 resin. On the third day, transfer the samples to embedding molds, add an appropriate amount of Epon812 resin, adjust the sample position as needed, and place the molds in a 60 °C oven for polymerization for 48 h. Prepare ultrathin sections (~ 70 nm) from the embedded samples and stain them with a 2% aqueous uranyl acetate solution at room temperature for 10-20 min, avoiding light exposure during staining. Stain the sections with lead citrate for 5-10 min, taking care to prevent CO₂ contamination during staining. After completing the staining process, the samples are ready for observation and imaging under the transmission electron microscope.

### Dual Luciferase Assay

For cell transfection followed by dual luciferase reporter assays, HEK293T cells or NIH 3T3 cells were co-transfected with PsiCHECK-2 plasmid constructs and OE-*Hilnc*/OE-*h-Hilnc*/pcDNA3.1(+) plasmids. 48 h post-transfection, cell lysates were harvested, and dual luciferase activity was measured with Dual-Luciferase® Reporter Assay System (Promega, E1910) according to the manufacturer's instructions, using a GloMax 20/20 Luminometer (Promega).

For *in vitro* translation followed by dual luciferase reporter assays, *in vitro* protein synthesis was performed using the TnT® Quick Coupled Transcription/Translation System (Promega, L1170), according to the standard protocol provided by Promega. The reaction mixture was supplied with *in vitro* synthesized *Hilnc* RNA. After protein synthesis, the reaction mixtures were directly subjected to the same luminescence detection procedures as those used for cell lysates.

### *In vitro* RNA Synthesis

To synthesize biotin-labeled RNA molecules *in vitro*, we used the RNAmax-T7 Biotin Labeling Transcription Kit (RiboBio, C11002-1). The synthesized RNA was purified and subsequently used for RNA pull-down and other experiments. The procedure followed the standard methods provided by Ribobio. The DNA linear template for the target fragment was designed to include the T7 promoter sequence at the 5' end (5' TAATACGACTCACTATA**G**GG 3'). Transcription initiated from the bold “G”, ensuring that the synthesized RNA did not contain any unintended sequences. For unbiotinylated RNA synthesis, the Biotin labelling mix in the recipe was substituted with normal rNTPs (Promega, E601D, E602D, E603D, E604D).

### RNA Pull-down Assays

For RNA-RNA pull-down, 500ng each of synthesized biotinylated bait and unbiotinylated prey were mixed in 200μL pull-down lysis buffer (20 mM Tris-HCl, pH 7.5, 100 mM KCl, 5 mM MgCl_2_, 0.3% NP-40) supplemented with 1U/μL RNase inhibitor (MCE, HY-K1033) and incubated on orbital shaker at 60 rpm at room temperature for 1 h. For RNase treatment, 2μg/μL RNase A (NEB, T3018L), 0.1U/μL RNase III (NEB, M0245S), or 0.2U/μL RNase H (NEB, M0297S), together with the corresponding RNase buffer, was added to the RNA mix, respectively. The mix were then incubated at 37 °C for 1 h. 20μL streptavidin magnetic beads (MCE, HY-K0208) were washed three times in the pull-down lysis buffer, then added to the RNA-RNA mix and pulled down by magnetic enrichment, followed by RT-qPCR for detection of the prey RNAs.

For RNA pull down of proteins from cell lysates, 1μg of biotin-labeled RNA was heated to 90 °C for 2 min to disrupt secondary structures and then placed on ice for 2 min to allow proper secondary structure formation. Cells (2 × 10 ^ 7) were lysed in pull-down lysis buffer (20 mM Tris-HCl (pH 7.5), 100 mM KCl, 5 mM MgCl_2_, 0.3% NP-40) supplemented with complete protease inhibitor cocktail and 1 U/µL RNase inhibitor, then sonicated and centrifuged until the cell lysate was clear. 5% of the lysate were prepared for input sample, and the rest of the lysate was pre-treated with 10 µg/mL yeast tRNA (Invitrogen, AM7119) and 50 µL of streptavidin-conjugated beads (MCE, HY-K0208) by rotating at 4 °C for 30 min. Folded RNA was then added to the pre-cleared cell lysate and rotated at 4 °C for 1 h. Next, 50 µL of washed streptavidin-conjugated beads were added to the reaction and incubated at 4 °C for 4 h. Then the beads were washed four times with pull-down lysis buffer (20 mM Tris-HCl (pH 7.5), 100 mM KCl, 5 mM MgCl_2_, 0.3% NP-40) supplemented with complete protease inhibitor cocktail and 1 U/µL RNase inhibitor. Proteins bound to the beads were separated by SDS-PAGE and subjected to Western blotting (WB) or mass spectrometry analysis.

### RNA Fluorescence *In Situ* Hybridization (FISH)

Fluorescently labeled single-stranded nucleic acids were used as probes to specifically hybridize with the target RNA based on base pairing principles. After denaturation, annealing, and renaturation, the hybridization complex of the target RNA and the nucleic acid probe was formed. The target RNA was then subjected to localization analysis using a fluorescence microscope. The probe synthesis was performed by GenePharma. The positive control probe was mouse 18S rRNA (5' CY3), provided by GenePharma.

*Hilnc* Probes (5' FAM-conjugated):

Probe 1: GTCTCCAGGCGAGGTGATTG

Probe 2: ATCAAGAAATCAAAGTACGCAGT

Probe 3: AAGAATCCAAGAATAAGGTGACAGT

*Ucp1*-3'UTR Probes (5' CY3-conjugated):

Probe 1: ATGTTCAGTATCTCTTCCTCCAAGT

Probe 2: CTGGAAATGATCTTGTAATGTAAAT

Probe 3: CCAAAGACCAGTTAAATACAGGACT

For FISH on tissue sections, the RNA FISH kit (GenePharma) was used, and the experiment was conducted according to the standard protocol provided by GenePharma. For FISH on cells, SVF cells were plated in multi-well chamber slides (BD Falcon) and allowed to differentiate. After the cells have completed differentiation, the cells were fixed and processed using the same procedure as for tissue sections. Images were acquired using Leica TCS SP8 confocal microscope.

### Mass Spectrometry of RNA Pull-Down Proteins

RNA pull-down beads were resuspended in 50 µL of lysis buffer (2% SDS, 100 mM Tris, 100 mM DTT, pH 7.6), heated at 95 °C for 10 min, and then centrifuged to collect the supernatant. For protein digestion using the SP4 method, 50 µL of sample and 20 µL of glass beads (1000 µg) were resuspended in 100 µL of re-suspension buffer (2 M urea acid (UA), 5 mM Tris, pH 7.6). 400 µL of acetonitrile was added and mixed, followed by centrifugation at 16,000 × g for 5 min at 4 °C to remove the supernatant. The pellet was washed four times with 1 mL of 80% ethanol, removing the supernatant each time. 100 µL of trypsin solution (10 ng/µL) was added, and the samples were incubated at 37 °C for 17 h for enzymatic digestion. The next day, samples were centrifuged at 15,000 × g for 5 min, and the supernatant was collected, lyophilized, desalted, and lyophilized again. Peptides were resuspended in 15 µL of 0.1% formic acid solution, centrifuged at 12,000 × g for 5 min, and the supernatant was transferred to vials for mass spectrometry analysis using a Q Exactive HF mass spectrometer (Thermo Scientific). Data were searched against a protein database using MaxQuant (version 1.6.17.0), with a false discovery rate (FDR) for protein identification controlled to be less than 1%.

### RNA Immunoprecipitation (RIP)

Cells were harvested and lysed in NT2 buffer (50 mM Tris-HCl pH 7.4, 150 mM NaCl, 1 mM MgCl₂, 0.05% NP-40) supplemented with RNase inhibitor (1 U/µL) (MCE, HY-K1033) and 1X protease inhibitor cocktail (Targetmol, C0001). Mouse iWATs were collected post-euthanasia and homogenized in ice-cold NT2 buffer containing 1U/µL RNase inhibitor and protease inhibitor cocktail using a tissue lyser (Bead Ruptor 24 Elite, OMNI international) for 20 seconds. Lysates were centrifuged at 4 °C, 14,000 × g for 10 min, and the supernatant was transferred to fresh 2 mL tubes. Total protein was quantified using the Pierce BCA Protein Assay Kit (Thermo Scientific), and 3 mg of cytoplasmic lysate protein was used for immunoprecipitation.

Anti-IGF2BP2 antibody (Proteintech, 82757-2-RR) and normal rabbit IgG (Proteintech, B900610) were added to the clarified lysate and incubated at 4 °C for 1 h. Then, 30 µL of Protein A/G magnetic beads (MCE, HY-K0202) were washed in NT2 buffer twice and added to the samples, incubated at 4 °C overnight. Beads were washed three times with RIP wash buffer (50 mM Tris-HCl pH 7.4, 455 mM NaCl, 1 mM MgCl_2_, 0.09% NP-40, 0.5% Sodium Deoxycholate, 1X protease inhibitor cocktail, RNase inhibitor (1 U/µL)). Bound RNAs were isolated from the beads using Trizol (Invitrogen) according to the manufacturer's instructions. The RNA component was analyzed by RT-qPCR.

### Adenovirus Infection of SVF Cells

The adenoviral vector used was Adeasy015-pAdEasy-EF1-MCS-CMV-mCherry. The expression plasmids were constructed by inserting the sequences of Full-length *Hilnc*, *HilncΔ450-600*, and *Hilnc450-600nt* into the adenoviral vector. The construction of adenoviral plasmids, viral amplification and concentration, as well as titer determination, were performed by Hanbio. Infection procedures followed the standard methods provided by Hanbio, with a multiplicity of infection (MOI) of 100. Infection efficiency was assessed using mCherry fluorescence and confirmed by qPCR detection of the overexpressed fragments.

### Molecular Docking

The binding mode between the protein and RNA was predicted through an integrated computational approach. The process began with the prediction of the RNA's secondary structure (http://rna.tbi.univie.ac.at/cgi-bin/RNAWebSuite/RNAfold.cgi). The predicted secondary structure was used as input to generate a three-dimensional model of the RNA (http://biophy.hust.edu.cn/new/3dRNA/create) 43. The generated 3D model of the RNA was then used alongside the protein structure (UniProt) in a molecular docking simulation to determine the optimal complex formation and assess binding affinity (HDOCK Server) 44.

### Statistical Analyses

Statistical analyses were performed using GraphPad Prism (v10.0) and R (3.4.0). Data are presented as Mean ± SEM unless otherwise specified. Statistical significance was determined using the following tests: Unpaired t-test: Used for comparisons between two groups. One-way ANOVA: Used for comparisons among multiple groups, followed by Tukey's multiple comparisons test. Two-way ANOVA: Used for experiments with two independent variables, followed by Bonferroni's multiple comparisons test. Pearson Correlation Coefficient: Used to assess the correlation between two continuous variables. ANCOVA 45: used for assessing the effect of genotype on body weight, with initial body weight included as a covariate. Preceded by a test for homogeneity of variances, with partial eta-squared (η²p) calculated to quantify the effect size, respectively.

P-values < 0.05 were considered statistically significant. Specific statistical tests and sample sizes are indicated in the figure legends and tables.

## Results

### Adipose-tissue-specific *Hilnc* knockout leads to altered iWAT morphology and energy metabolism in mice

**Figure 1:**
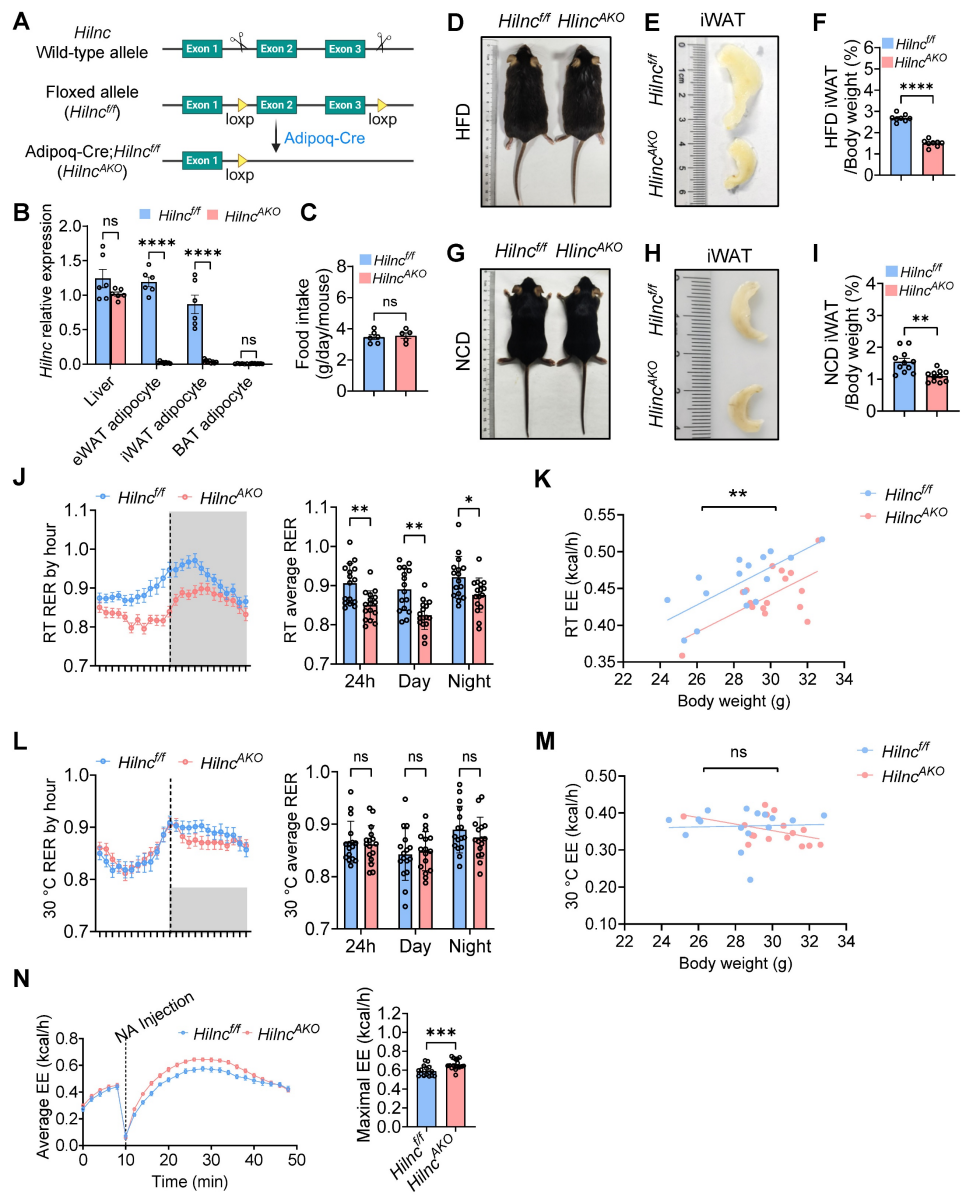
** Adipose-tissue-specific *Hilnc* knockout leads to altered iWAT morphology and energy metabolism. A)** Strategy of construction of *Hilnc^f/f^* mice and generation of *Adipoq-Cre; Hilnc^f/f^* (*Hilnc^AKO^*) mice. **B)** RT-qPCR results of *Hilnc* knock-out efficiency in liver and adipocytes of *Hilnc^f/f^* and *Hilnc^AKO^* mice (N = 3 technical replicates of 2-3 mice from each strain). **C)** Comparison of food intake between NCD-fed *Hilnc^f/f^* and *Hilnc^AKO^* mice (N = 6 mice for *Hilnc^f/f^* and N = 5 mice for *Hilnc^AKO^*). **D)** Whole-body size of HFD-fed *Hilnc^f/f^* and *Hilnc^AKO^* mice. **E-F)** Size of iWAT (E) and iWAT weights as percentage of whole-body weights (F) of HFD-fed *Hilnc^f/f^* and *Hilnc^AKO^* mice (N = 8 mice for each strain). **G)** Whole-body size of NCD-fed *Hilnc^f/f^* and *Hilnc^AKO^* mice. **H-I)** Size of iWAT (H) and iWAT weights as percentage of whole-body weights (I) of NCD-fed *Hilnc^f/f^* and *Hilnc^AKO^* mice (N = 11 mice for each strain). **J)** RER per hour (left) and day-vs-night breakdown (right) between RT NCD-fed *Hilnc^f/f^* and *Hilnc^AKO^* mice (N = 16 mice for each strain). **K)** 24h overall energy expenditure (EE) between RT NCD-fed *Hilnc^f/f^* and *Hilnc^AKO^* mice (N = 16 mice for each strain). **L)** RER per hour (left) and day-vs-night breakdown (right) between 30 °C NCD-fed *Hilnc^f/f^* and *Hilnc^AKO^* mice (N = 16 mice for each strain). **M)** 24h overall energy expenditure (EE) between 30 °C NCD-fed *Hilnc^f/f^* and *Hilnc^AKO^* mice (N = 16 mice for each strain). **N)** Overall energy expenditure over time (left) and comparison of maximal energy expenditure (right) of norepinephrine (NA)-injected RT NCD-fed *Hilnc^f/f^* and *Hilnc^AKO^* mice (N = 16 mice for *Hilnc^f/f^* and N = 17 mice for *Hilnc^AKO^*). Data are presented as mean ± SEM. Student's t-tests were used for statistical analysis (For B, C, F, I). ANCOVA were used for statistical analysis (for J, K, L, M, N), adjusted for body weight, genotype effect is independent of body weight. (ns: not significant, *: *p <* 0.05, **: *p <* 0.01, ***: *p <* 0.001, ****: *p <* 0.0001).** See also Figure S1.**

*Hilnc* has previously been shown to play an important role in regulating lipid metabolism in the liver 34. We observed that the body size of high-fat-diet (HFD)-fed *Hilnc^-/-^* mice were smaller compared to that of the wild-type (WT) mice (Figure S1A), and the volumes of various adipose tissues, including epididymal white adipose tissue (eWAT), inguinal white adipose tissue (iWAT), and brown adipose tissue (BAT), were reduced in HFD-fed *Hilnc^-/-^* mice (Figure S1B-D), with no discernible difference in food intake (Figure S1E). This prompted the question of whether *Hilnc* has a physiological role in adipose tissues. To explore this, we first assessed the expression of *Hilnc* in adipocytes using reverse transcription quantitative polymerase chain reaction (RT-qPCR). RT-qPCR results demonstrated that *Hilnc* was predominantly expressed in eWAT and iWAT adipocytes, with expression levels comparable to that of the liver, but not in BAT adipocytes (Figure S1F). To specifically knockout *Hilnc* in adipocytes, we generated *Hilnc^f/f^* mice by inserting loxP sequences flanking exons 2 and 3 of the *Hilnc* locus (Figure 1A). These *Hilnc^f/f^* mice were subsequently crossed with *Adipoq-Cre* mice to generate adipocyte-specific *Hilnc* knockout mice (*Adipoq-Cre; Hilnc^f/f^* mice, hereafter referred to as *Hilnc^AKO^* mice) (Figure 1A, Figure S1G). The successful knockout of *Hilnc* in eWAT and iWAT was validated by RT-qPCR (Figure 1B).

Next, we investigated whether the specific knockout of *Hilnc* in adipocytes affected adipose tissue characteristics. Again, we observed that there were no significant differences in food intake between the two groups (Figure 1C). No significant differences in body size (Figure 1D), gross body weight, or fat content (Figure S1H) were observed between HFD-fed *Hilnc^AKO^* mice and *Hilnc^f/f^* mice, nor were there any notable changes in eWAT and BAT morphology and their weight proportions between the two mouse strains (Figure S1I-J). However, the volume and weight of iWAT in *Hilnc^AKO^* mice were significantly smaller compared to *Hilnc^f/f^* mice (Figure 1E-F), indicating that iWAT was particularly affected by the loss of *Hilnc* in adipocytes. In mice fed a normal chow diet (NCD), similar to HFD-fed mice, no significant changes in body weight, fat content, or eWAT and BAT morphology and their weight proportions (Figure 1G, Figure S1K-M) were observed. However, the volume and weight of iWAT were significantly decreased in *Hilnc^AKO^* mice (Figure 1H-I), indicating that these changes in iWAT are specifically attributed to the adipocyte-specific loss of *Hilnc*.

To examine whether the knockout of *Hilnc* in adipocytes impacts overall mouse metabolism, we analyzed the energy metabolism profiles of NCD-fed *Hilnc^f/f^* and *Hilnc^AKO^* mice using metabolic cages. While no notable differences were detected in overall movement characteristics between the two groups (Figure S1N), *Hilnc^AKO^* mice exhibited a significantly lower respiratory exchange ratio (RER) (Figure 1J) alongside reduced energy expenditure (EE) (Figure 1K) when housed at room temperature (RT). This decrease in RER indicates a systemic shift in energy substrate preference, with *Hilnc^AKO^* mice favoring lipid oxidation, whereas *Hilnc^f/f^* controls maintained a metabolic profile characteristic of predominant carbohydrate utilization.

The observed difference in EE under these conditions prompted further investigation. Since standard room temperature (22 °C) imposes a chronic cold stress on mice, which can elevate basal energy consumption, we sought to determine whether the metabolic disparity was stress-dependent. To test this, we acclimated mice to thermoneutral conditions (30 °C) for two weeks prior to metabolic assessment. At thermoneutrality, the difference in EE between *Hilnc^AKO^* and *Hilnc^f/f^* mice was abolished (Figure 1M). Furthermore, although *Hilnc^AKO^* mice still displayed a trend toward lower RER during the dark phase, this difference was markedly attenuated and lost statistical significance (Figure 1L).

The emergence of the EE phenotype specifically at room temperature, coupled with the persistent shift in RER and observed morphological changes in iWAT, led us to hypothesize that *Hilnc^AKO^* mice might possess an enhanced metabolic capacity. To test this, we maximally activated non-shivering thermogenesis via norepinephrine (NA) injection in RT-housed mice. In contrast to the unstimulated state, this challenge revealed a significantly higher peak energy expenditure in *Hilnc^AKO^* mice compared to controls (Figure 1N), demonstrating their superior maximal thermogenic capacity.

In summary, the loss of *Hilnc* in adipocytes specifically leads to changes in iWAT morphology, which is associated with altered energy metabolism and a shift in preferred energy substrates.

### The loss of *Hilnc* in adipose tissues promotes iWAT beiging and thermogenesis

**Figure 2:**
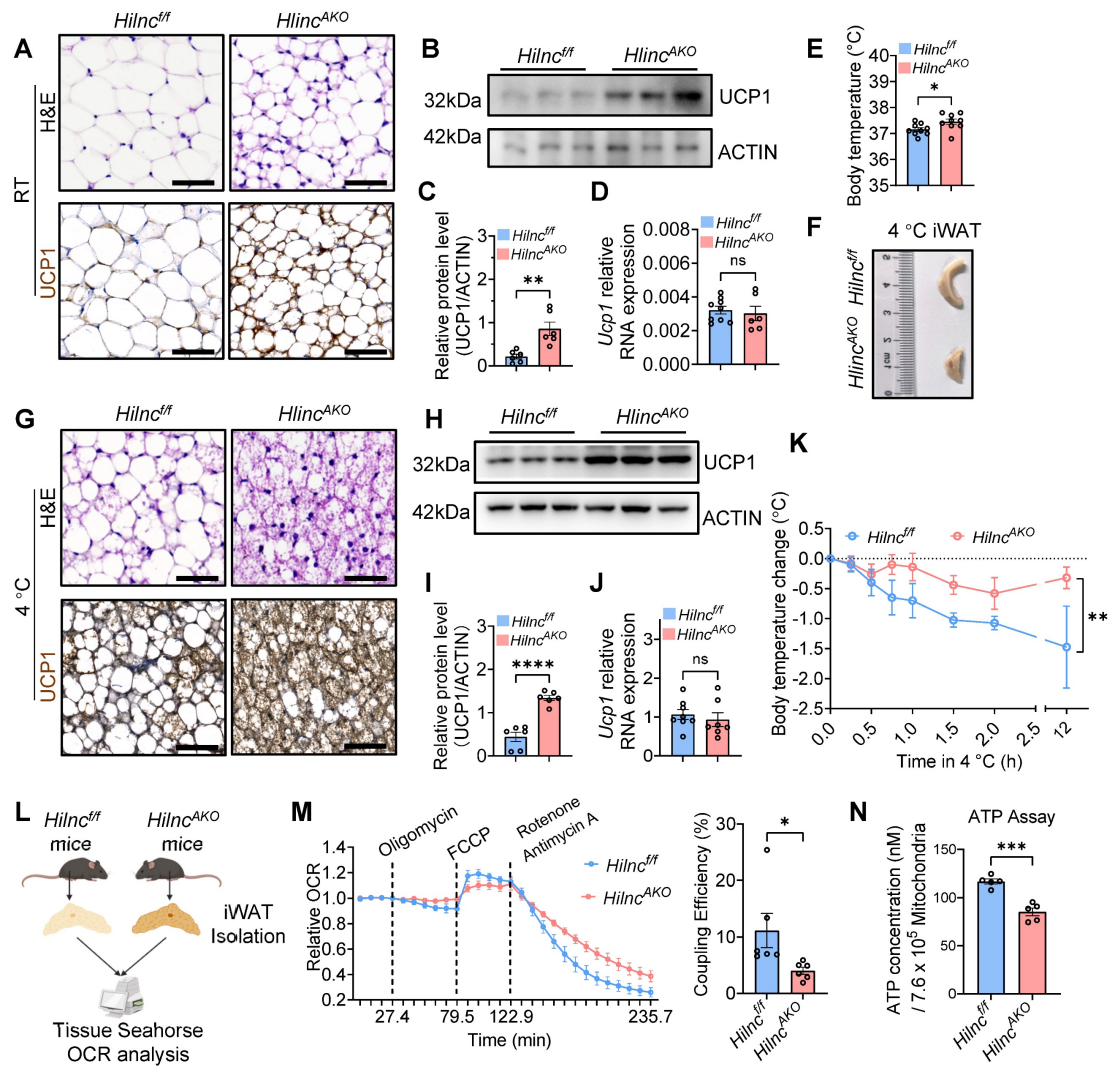
** The loss of *Hilnc* in adipose tissues promotes iWAT beiging and thermogenesis. A)** H&E stain and UCP1 immunohistochemistry (IHC) of RT *Hilnc^f/f^* and *Hilnc^AKO^* iWATs. Scale bar = 50 μm. **B)** Representative UCP1 Western blots of RT *Hilnc^f/f^* and *Hilnc^AKO^* iWAT homogenates. **C)** Western blots quantifications of RT *Hilnc^f/f^* and *Hilnc^AKO^* iWAT homogenates (N = 6 mice for each strain). **D)** RT-qPCR analysis of *Ucp1* mRNA expression in iWAT from RT-maintained* Hilnc^f/f^* and *Hilnc^AKO^* mice (n = 9 mice for *Hilnc^f/f^* and n = 6 mice for *Hilnc^AKO^*). **E)** Rectal temperatures of RT *Hilnc^f/f^* and *Hilnc^AKO^* mice (N = 9 mice for each strain). **F)** Size comparison of iWATs between cold-acclimated RT *Hilnc^f/f^* and *Hilnc^AKO^* mice. **G)** H&E stain and UCP1 immunohistochemistry (IHC) of cold-acclimated *Hilnc^f/f^* and *Hilnc^AKO^* iWATs. Scale bar = 50 μm. **H)** Representative UCP1 Western blots of cold-acclimated *Hilnc^f/f^* and *Hilnc^AKO^* iWAT homogenates. **I)** Western blots quantifications of cold-acclimated *Hilnc^f/f^* and *Hilnc^AKO^* iWAT homogenates (N = 6 mice for each strain). **J)** RT-qPCR analysis of *Ucp1* mRNA expression in iWAT from cold-acclimated* Hilnc^f/f^* and *Hilnc^AKO^* mice (n = 8 mice for *Hilnc^f/f^* and n = 7 mice for *Hilnc^AKO^*). **K)** Average changes in rectal temperature of cold-acclimated *Hilnc^f/f^* and *Hilnc^AKO^* mice upon re-exposure to cold (N = 4 mice for *Hilnc^f/f^* and N = 5 mice for *Hilnc^AKO^*). **L)** Diagram of iWAT isolation for OCR analysis by Seahorse analyzer. **M)** Compiled relative changes of iWAT OCRs during the Seahorse program (left), and coupling efficiencies, calculated as ((OCR_ATP production_ / OCR_Basal respiration_) × 100%) (right) between cold-acclimated *Hilnc^f/f^* and *Hilnc^AKO^* iWATs (N = 6 biological replicates). **N)** Quantification of total ATP released from an equal number of isolated mitochondria from intact iWAT of *Hilnc^f/f^* and *Hilnc^AKO^* mice (N = 5 biological replicates). Data are presented as mean ± SEM. Student's t-tests were used for statistical analysis (C, D, E, I, J, K, M and N). (ns: not significant, *: *p <* 0.05, **: *p <* 0.01, ***: *p <* 0.001, ****: *p <* 0.0001).** See also Figure S2 and Figure S3.**

To further characterize the effects of *Hilnc* on iWAT, we examined tissue histology in NCD-fed mice under room temperature (RT). Hematoxylin and eosin (H&E) staining revealed a marked reduction in adipocyte size and an increased presence of smaller, multilocular adipocytes in the iWAT of *Hilnc^AKO^* mice compared to *Hilnc^f/f^* controls. The emergence of these multilocular adipocytes was especially prominent within the apex region of the iWAT (Figure 2A, Figure S2A). Notably, iWAT can undergo beiging under low temperatures, a process during which mitochondrial UCP1 is upregulated in adipocytes, contributing to increased intracellular metabolic rate and thermogenesis to counteract heat loss 46, 47. We next assessed the expression of UCP1 to further evaluate the extent of adipocyte beiging. UCP1 immunohistochemistry and Western blot analysis demonstrated that, compared to *Hilnc^f/f^* controls, *Hilnc^AKO^* mice exhibited spontaneous upregulation of UCP1 protein and enhanced beiging of iWAT, even under RT conditions (Figure 2A-C, Figure S2B). However, *Ucp1* mRNA levels remained unchanged between the two groups (Figure 2D), suggesting that *Hilnc* does not regulate *Ucp1* transcription but may instead influence UCP1 protein stability or translation process. Given that UCP1 upregulation and iWAT beiging are closely linked to thermogenesis, we next examined the impact of *Hilnc* deletion on thermogenic function. *Hilnc^AKO^* mice exhibited significantly higher rectal temperatures compared to *Hilnc^f/f^* controls (Figure 2E), indicating enhanced thermogenic activity in the regular state.

To further investigate the role of *Hilnc* in iWAT beiging, we exposed *Hilnc^f/f^* and *Hilnc^AKO^* mice to cold conditions. Both groups exhibited significant iWAT beiging during cold acclimation compared to mice maintained at RT. iWATs from *Hilnc^AKO^* mice were smaller (Figure 2F), displayed a denser histological architecture, and showed higher UCP1 protein levels (Figure 2G-I, Figure S2C-D) compared to *Hilnc^f/f^* controls. Consistent with observations under RT conditions, *Ucp1* mRNA levels remained unchanged between the two groups after cold exposure (Figure 2J), further suggesting that *Hilnc* regulates UCP1 expression at a post-transcriptional level. To assess cold adaptation, mice were returned to RT before re-exposure to low temperature. *Hilnc^AKO^* mice maintained significantly higher core body temperatures than *Hilnc^f/f^* mice under cold stress (Figure 2K).

Given the clear differences in iWAT, we sought to determine whether the increased thermogenesis was solely attributable to iWAT. Contacting the result mentioned above, NA injection can stimulate lipolysis and activation of beige and brown adipocytes, although iWAT was the primary site of interest in this study, BAT is also known to play a pivotal role in thermogenic regulation and overall energy metabolism. We therefore first examined the potential contribution of BAT, the primary adipose tissue responsible for thermogenesis. The size, histological architecture, and UCP1 protein levels of BAT were comparable between WT and *Hilnc^-/-^* mice under RT or cold-acclimated conditions (Figure S3A-H). Similarly, no significant differences were observed in eWAT between the two groups (Figure S3I). Since our conditional knockout model utilizes Adiponectin-promoter-driven Cre expression, *Hilnc* is also deleted in mature adipocytes of eWAT. We therefore investigated whether *Hilnc* ablation affects the metabolic characteristics of eWAT mature adipocytes in *Hilnc^AKO^* mice compared to* Hilnc^f/f^* controls. We analyzed the expression of key genes involved in lipid synthesis, transport, breakdown, and fatty acid uptake. The results indicated that *Hilnc* knockout did not induce significant alterations in lipid metabolism pathways in eWAT (Figure S3J). These results suggest that the impact of *Hilnc* knockout is not prominently manifested in white adipocytes, which further prompts us to reconsider the physiological distinctions between iWAT and eWAT and underscores the heterogeneity of *Hilnc*'s physiological functions in these two adipose depots. To further assess the specific contribution of BAT, we generated BAT-specific *Hilnc* knockout mice (*Ucp1-Cre; Hilnc^f/f^*, hereafter referred to as *Hilnc^UKO^*) by crossing *Hilnc^f/f^* mice with *Ucp1-Cre* mice (Figure S3K). No significant differences were found in BAT morphology, UCP1 protein levels, or cold tolerance between *Hilnc^f/f^* and *Hilnc^UKO^* mice under either RT or cold-acclimated conditions (Figure S3L-T). These results suggest that *Hilnc* plays a minimal role in regulating BAT function under both basal and cold-induced conditions, and that the enhanced thermogenic phenotype observed in *Hilnc*-deficient mice is primarily attributable to changes in iWAT.

To further investigate the direct contribution of iWAT to thermogenesis, we isolated iWAT from cold-acclimated *Hilnc^f/f^* and *Hilnc^AKO^* mice and analyzed tissue respiration levels using a Seahorse analyzer (Figure 2L). Although no significant differences in basal respiration rate and maximum respiration rate were observed under stress conditions (Figure S2U-V), *Hilnc^AKO^* iWAT exhibited a strong trend of decreased ATP production rate (Figure S2W), leading to a significant decrease in coupling efficiency (Figure 2M). To further confirm that the differences in coupling efficiency and ATP production were linked to the elevated UCP1 expression on mitochondria, we isolated mitochondria from iWAT of mice subjected to the 4 °C acclimation and measured the ATP content normalized to an equal number of mitochondria. The results showed that mitochondria isolated from the iWAT of *Hilnc^AKO^* mice contained significantly lower ATP levels than those from *Hilnc^f/f^* mice (Figure 2N). This finding aligns with the energy metabolic profile observed at the tissue level in Seahorse assays, and the use of purified mitochondria served to accentuate the disparity in ATP generation. Since UCP1 is located in the mitochondrial inner membrane, its activity causes proton leakage, which significantly diminishes ATP synthesis efficiency.

In summary, the loss of *Hilnc* in adipose tissue results in increased UCP1 protein levels in iWAT, which directly contributes to enhanced thermogenesis and improved body temperature maintenance in *Hilnc^AKO^* mice.

### Loss of *Hilnc* in Mature Adipocytes Enhances Mitochondrial Function

Given the considerable heterogeneity of iWAT tissue, we sought to identify the specific cell compartment responsible for the increased thermogenesis observed in *Hilnc^AKO^* mice. To determine whether *Hilnc*-associated UCP1 upregulation occurred during the differentiation of new adipocytes, we used flow cytometry to examine the stromovascular fraction (SVF), which contains SCA-1^+^ preadipocytes 48, 49. The frequency of SCA-1^+^ preadipocytes did not differ significantly between WT and *Hilnc^-/-^* mice, nor between *Hilnc^f/f^* and *Hilnc^AKO^* mice (Figure S4A-D). Additionally, RT-qPCR analysis revealed no significant differences in the expression levels of key transcription factors associated with adipocyte differentiation and beiging, including *Prdm16*, *Pgc1a*, *Cebpa*, and *Cidea* (Figure S4E). These findings suggested that *Hilnc* does not affect the frequency or differentiation program of the iWAT SVF. The qPCR analysis also confirmed that *Hilnc* expression was completely unaffected in the SVF of *Hilnc^AKO^* mice (Figure S4F) whereas *Hilnc* expression was significantly reduced in differentiated *Hilnc^AKO^* adipocytes (Figure S4G). Therefore, the *Hilnc^AKO^* phenotype firmly indicates that *Hilnc* does not function within the SVF, but rather exerts its role specifically in mature adipocytes.

**Figure 3:**
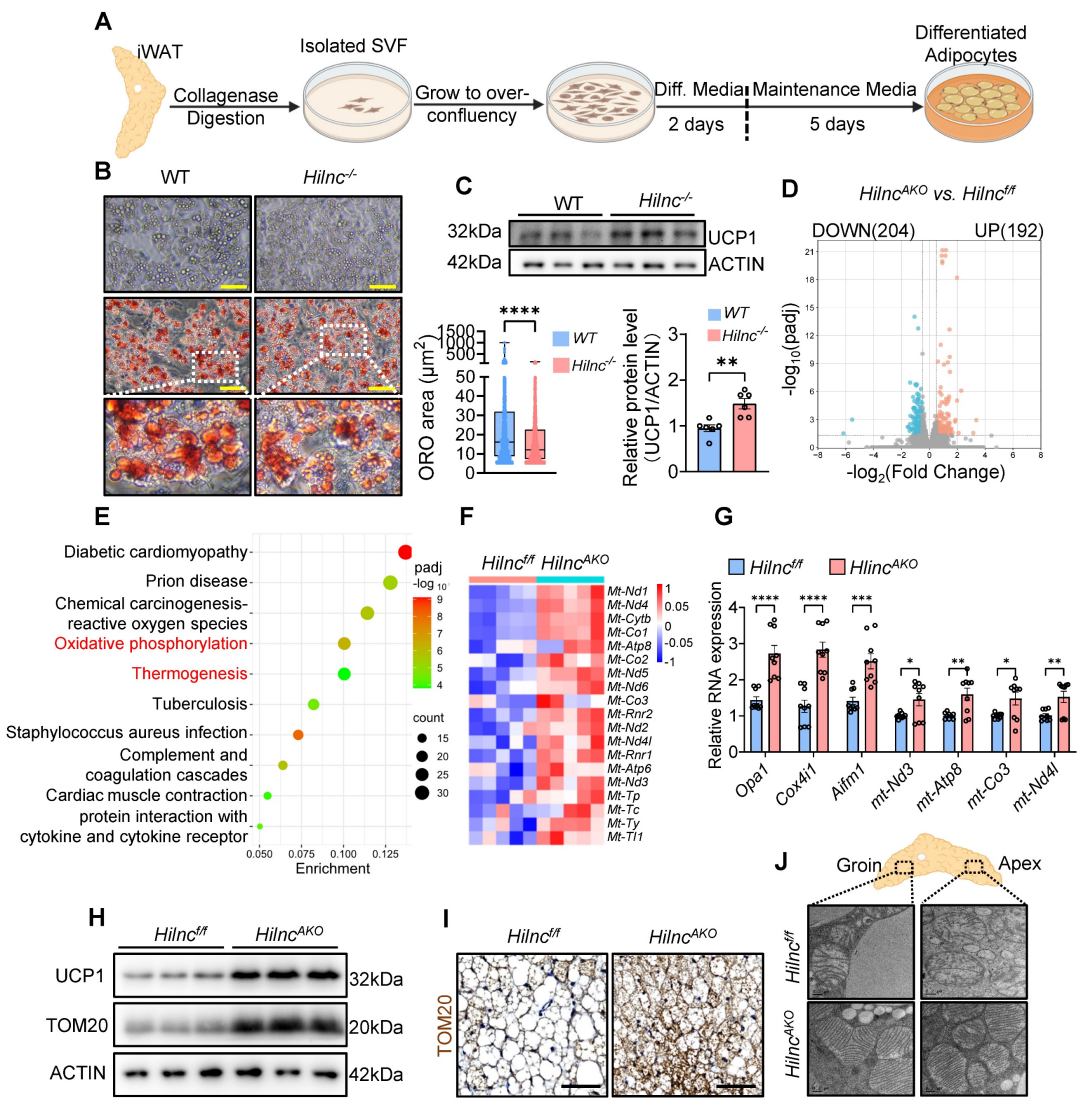
** The loss of *Hilnc* in mature adipocytes results in increased mitochondrial function. A)** Protocol of *in vitro* SVF isolation and differentiation into mature adipocytes. **B)** Representative bright field images (left-top), Oil-red O stain images (left-bottom) and quantification of lipid droplet area from Oil Red O Staining (right) of differentiated adipocytes from SVFs of WT or *Hilnc^-/-^* iWATs (Scale bar = 100 μm). **C)** UCP1 western blots (top) and quantification (bottom) of differentiated adipocytes from SVFs of WT or *Hilnc^-/-^* iWATs (N = 6 biological replicates for each strain). **D)** Volcano plot showing differentially expressed genes between cold-acclimated *Hilnc^f/f^* and *Hilnc^AKO^* iWAT mature adipocytes. **E)** KEGG enrichment analysis of differentially regulated gene sets in cold-acclimated *Hilnc^f/f^* vs *Hilnc^AKO^* iWAT mature adipocytes. **F-G)** Expression levels of mitochondrial function-related genes in cold-acclimated *Hilnc^f/f^* vs *Hilnc^AKO^* iWAT mature adipocytes, analyzed by RNA-seq (F, N = 5 mice for each strain) and RT-qPCR (G, N = 3 technical replicates from 3 mice for each strain). **H-I)** TOM20 Western blots (H, N = 3 mice for each strain) and IHC (I) of cold-acclimated iWATs from *Hilnc^f/f^* and *Hilnc^AKO^* mice. Scale bar = 50 μm for I). **J)** Representative TEM images of the groin and apex ends of iWATs from cold-acclimated *Hilnc^f/f^* and *Hilnc^AKO^* mice, showing individual mitochondria morphology. Scale bar = 0.2 μm. Data are presented as mean ± SEM. Student's t-tests with Holm-Šídák's multiple comparison correction were used for statistical analysis (H). (*: *p <* 0.05, **: *p <* 0.01, ***: *p <* 0.001, ****: *p <* 0.0001).** See also Figure S4.**

Next, we investigated whether the loss of *Hilnc* in mature adipocytes directly contributes to the increase in UCP1 protein levels and respiration. Given the relatively short duration of the cell differentiation cycle, we isolated SVF from WT and *Hilnc^-/-^* mice to ensure a sustained and significant difference in *Hilnc* expression levels between the two groups established as controls throughout the experiment. The adherent fraction was then subjected to *in vitro* differentiation into mature adipocytes (Figure 3A). Consistently, no differences in SVF fraction frequency among adherent fractions were observed between plated WT and *Hilnc^-/-^* SVF at the onset of differentiation (Figure S4H). Differentiated mature adipocytes from *Hilnc^-/-^* SVF exhibited smaller lipid droplets (Figure 3B) and higher UCP1 protein levels (Figure 3C), suggesting that the loss of *Hilnc* in mature adipocytes is responsible for the increase of UCP1 protein levels in tissue and the decrease in lipid accumulation.

To further investigate the cellular phenotypic changes associated with the loss of *Hilnc* in mature adipocytes, we performed RNA sequencing on mature adipocytes isolated from iWAT of cold-acclimated *Hilnc^f/f^* and *Hilnc^AKO^* mice. In *Hilnc^AKO^* iWAT adipocytes, 192 genes were upregulated, and 204 genes were downregulated compared to *Hilnc^f/f^* iWAT adipocytes (Figure 3D). Kyoto Encyclopedia of Genes and Genomes (KEGG) enrichment analysis of the differentially expressed gene sets revealed predominant associations with oxidative phosphorylation and thermogenesis (Figure 3E), strongly suggesting that mitochondrial function is altered in *Hilnc^AKO^* iWAT adipocytes. Notably, several genes of mitochondrial genome origin were significantly upregulated (Figure 3F). The increases in mitochondrial function-related genes in the iWATs of cold-acclimated mice were confirmed by RT-qPCR (Figure 3G), as well as TOM20 (an outer mitochondrial membrane protein) western blotting and IHC of iWAT sections (Figure 3H-I).

To directly examine mitochondrial morphology in iWAT, we prepared iWAT from cold-acclimated *Hilnc^f/f^* and *Hilnc^AKO^* mice for transmission electron microscopy (TEM). Mitochondria biogenesis occurs more intensively in the cytoplasmic part of *Hilnc^AKO^* adipocytes at both the groin and the apex (Figure S4I). High-magnification imaging revealed that mitochondria in adipocytes at both the groin and apex regions of iWAT displayed morphological differences between genotypes, with mitochondria from *Hilnc^f/f^* mice showing more disorganized structure and fewer cristae compared to those from *Hilnc^AKO^* mice (Figure 3J), indicative of higher mitochondria activity and consistent with the observation of higher basal respiration rate in *Hilnc^AKO^* iWATs.

Collectively, these results suggested that the loss of *Hilnc* in mature adipocytes specifically leads to a global increase in mitochondrial function, which explains the observed increase in respiration and thermogenesis 50

### Binding between *Hilnc*, IGF2BP2 and the 3'UTR of *Ucp1* mRNA

After identifying the role of *Hilnc* in mature adipocytes, we focused on investigating the mechanism by which *Hilnc* regulates UCP1 protein levels. Interestingly, *Ucp1* mRNA levels did not differ significantly between *Hilnc^f/f^* and *Hilnc^AKO^* iWAT under either RT or cold-acclimated conditions (Figure 2D, 2J), implying that *Hilnc* modulates UCP1 protein levels via a post-transcriptional mechanism. To explore this further, we used an *in vitro* adipocyte differentiation assay to assess UCP1 protein stability by adding cycloheximide (CHX) at the end of differentiation to halt *de novo* synthesis of UCP1. No significant difference in the half-life of UCP1 protein was observed between WT and *Hilnc^-/-^* differentiated adipocytes (Figure S5A), ruling out the possibility that *Hilnc* modulates UCP1 protein levels through a post-translational mechanism. The PPAR pathway is known to play an important role in adipogenesis and iWAT beiging, and *Hilnc* has been shown to regulate the PPARγ signaling pathway in the liver 34. However, no significant differences in *Pparg* or its downstream genes were observed in iWAT adipocytes (Figure S5B), suggesting that, unlike in the liver, *Hilnc* does not function through the PPARγ signaling pathway in adipocytes. Based on all the above findings, we shifted our focus to how *Hilnc* directly controls *Ucp1* mRNA translation.

**Figure 4:**
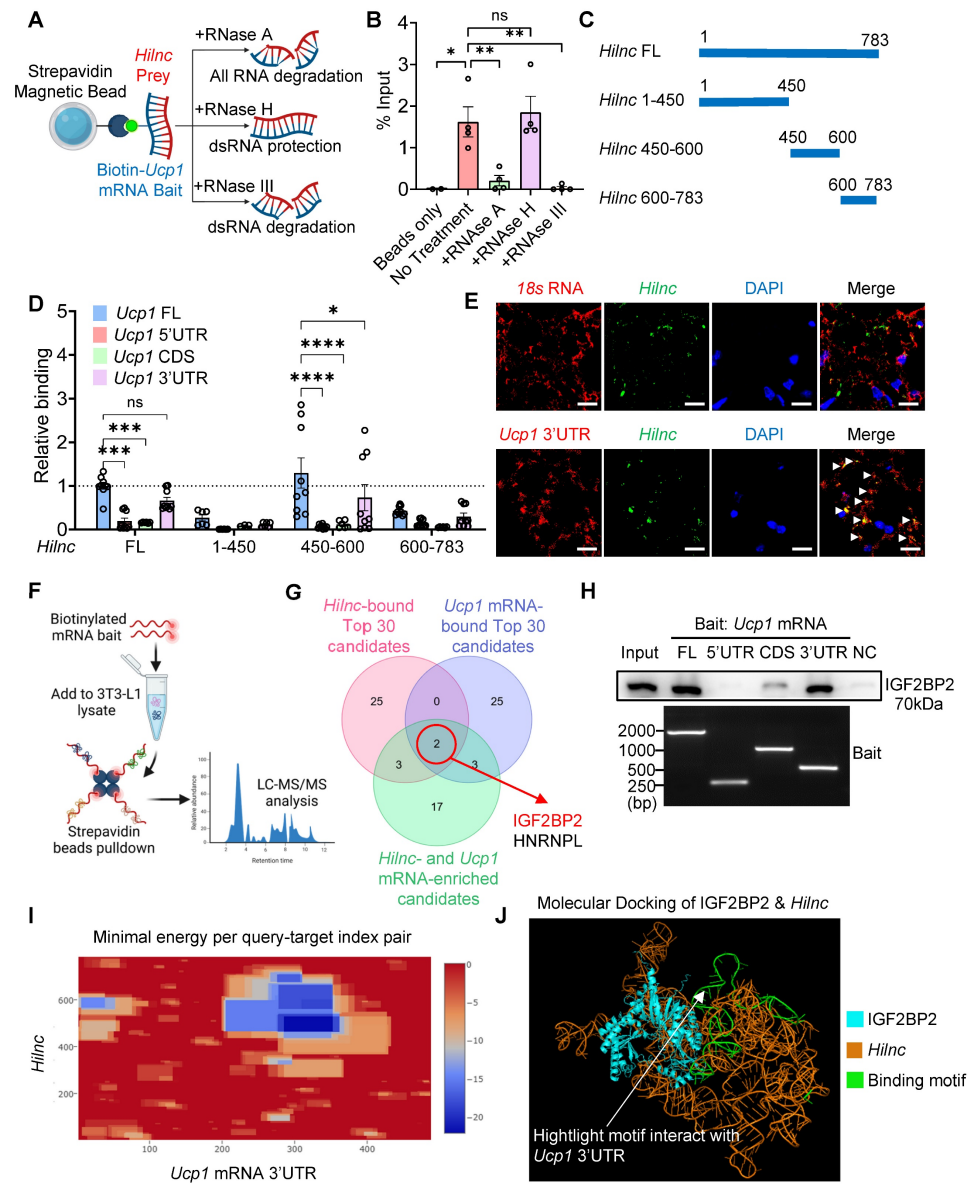
** Binding between *Hilnc*, IGF2BP2 and the 3'UTR of *Ucp1* mRNA. A)** Diagram of RNA-RNA binding experiment, followed by various RNase digestion to identify the RNA-RNA duplexes between *Hilnc* (Red) and *Ucp1* mRNA (Blue). **B)** RT-qPCR of *Hilnc* RNA abundance following RNase A, H or, III digestion of *in vitro Ucp1* mRNA pull-down products (N = 4 biological replicates). **C)** Diagram showing *Hilnc* segments used to identify the binding site between *Hilnc* and *Ucp1* mRNA. **D)** qRT-PCR analysis of parallel RNA-RNA binding experiments showing binding efficiencies between *Hilnc* segments and *Ucp1* mRNA segments (N = 3 technical replicates from 2-3 biological replicates). **E)** Fluorescent *in situ* hybridization (FISH) of 18S rRNA, *Ucp1* mRNA 3'UTR and* Hilnc* RNA in WT paraffin-embedded iWAT sections. Arrows indicate co-localization of fluorescent probes. Scale bar = 10 μm.** F)** Diagram of the biotinylated *Hilnc* and *Ucp1* mRNA pull-down experiments. **G)** Venn diagram showing the candidate proteins that binds strongly to *Hilnc* or *Ucp1* mRNA, and are enriched by both *Hilnc* and *Ucp1* mRNA. **H)** Western blot of IGF2BP2 (top) from the RNA pull-down experiment using biotinylated fragments of *Ucp1* mRNA as bait (bottom). **I)** *In silico* prediction of RNA-RNA interaction between *Hilnc* and *Ucp1* mRNA 3'UTR (https://rna.informatik.uni-freiburg.de/IntaRNA/Result.jsp). **J)** Molecular docking of IGF2BP2 & *Hilnc* (http://hdock.phys.hust.edu.cn/). The green highlight indicates the segment on *Hilnc* RNA with the most favorable (lowest) binding free energy to the *Ucp1* mRNA 3'UTR region. Data are presented as mean ± SEM. One-way ANOVA with Tucky's multiple comparison correction (B) and 2-way ANOVA with Dunnett's multiple comparison correction (D) was used for statistical analysis. (ns: not significant, *: *p <* 0.05, **: *p <* 0.01, ***:* p <* 0.001, ****: *p <* 0.0001).** See also Figure S5 and Tables S1-2.**

LncRNAs modulate cellular gene expression through various mechanisms, often requiring direct binding to target mRNA molecules 31. To investigate whether *Hilnc* binds directly to *Ucp1* mRNA, we used synthesized biotin-conjugated *Ucp1* mRNA as bait to pull down synthesized *Hilnc* RNA. The resulting complex was subjected to digestion with RNases of varying specificity, followed by RT-qPCR to confirm the formation of RNA-RNA duplexes (Figure 4A). The pull-down product was readily digested by RNase A and RNase III, which digest total RNA and double-stranded RNA, respectively. However, RNase H, which specifically degrades RNA in an RNA-DNA duplex, failed to digest the enriched *Hilnc*, suggesting successful binding between *Ucp1* mRNA and *Hilnc* RNA (Figure 4B). These results indicated that *Hilnc* forms an RNA-RNA duplex with *Ucp1* mRNA. To determine the specific binding site between *Hilnc* RNA and *Ucp1* mRNA, we conducted parallel RNA pull-down experiments with synthesized biotin-conjugated fragments of* Ucp1* mRNA and unconjugated *Hilnc* RNA fragments (Figure 4C). The 450-600nt region of *Hilnc* bound to the 3'UTR of *Ucp1* mRNA with similar efficiency as full-length (FL) *Hilnc* RNA binding to FL *Ucp1* mRNA (Figure 4D), revealing the primary binding site between the two RNA molecules. Additionally, we performed fluorescence *in situ* hybridization (FISH) with *Hilnc* and *Ucp1* mRNA 3'UTR probes in WT mouse iWAT sections. In contrast to *18s* rRNAs, which did not co-localize with *Hilnc* RNA, *Ucp1* mRNA 3'UTR and *Hilnc* RNA co-localized in adipocytes (Figure 4E).

To determine whether *Hilnc* directly affected *Ucp1* mRNA translation, we used a dual-luciferase reporting system: utilizing the psiCHECK-2 plasmid with simultaneous expression of firefly luciferase (Fluc) and renilla luciferase (Rluc), the 3'UTR sequence of *Ucp1* mRNA was cloned after Rluc (psiCHECK2-Rluc-*Ucp1*-3'UTR; Figure S5C), and the plasmid was subjected to *in vitro* transcription and translation by rabbit reticulocyte lysate, with or without addition of synthesized *Hilnc* RNA. We hypothesized that the ratio between Rluc and Fluc luminescence should decrease if *Hilnc* directly suppresses translation via binding to the 3'UTR of *Ucp1* mRNA. However, we detected no significant difference in Rluc *in vitro* translation efficiency (Figure S5D), suggesting that *Hilnc* does not directly suppress *Ucp1* mRNA translation. One of the mechanisms by which lncRNAs modulate protein translation is by guiding translation-suppressing protein to mRNAs through RNA-RNA binding 31, 51. Therefore, we attempted to identify protein modulators of *Ucp1* mRNA translation that may be associated with *Hilnc*. We used biotin-conjugated *Ucp1* mRNA or *Hilnc* RNA as bait to pull down interacting proteins from 3T3-L1 lysates, followed by analysis using liquid chromatography with tandem mass spectrometry (LC-MS/MS) (Figure 4F). The enriched proteins were then overlapped between the two bait RNAs, allowing us to discover potential candidates that might facilitate *Hilnc*-mediated translational blockade of *Ucp1* mRNA. Among the proteins that are highly bound to either RNA and enriched by both RNAs (Figure 4G, Table S1-3), insulin-like growth factor 2 mRNA-binding protein 2 (IGF2BP2) emerged as one of the two promising candidates. The other candidate, heterogeneous nuclear ribonucleoprotein L (HNRNPL), is known to play pivotal roles in mRNA splicing and stability 52, but little is known about whether it directly affects mRNA translation efficiency. On the other hand, others have shown that IGF2BP2 regulates UCP1 translation through the UTRs of *Ucp1* mRNA 28, and our previous research has indicated that IGF2BP2 binds to *Hilnc* and is involved in lipid metabolism in the liver 34. Therefore, IGF2BP2 is likely involved in *Hilnc*-mediated attenuation of *Ucp1* mRNA translation, and was warranted for further investigation. The interaction between IGF2BP2 and *Hilnc* was validated by RNA immunoprecipitation (RIP) of IGF2BP2 followed by RT-qPCR of *Hilnc* (Figure S5E). To identify the specific interaction site on *Ucp1* mRNA, synthesized biotin-conjugated *Ucp1* mRNA fragments were used in an RNA pull-down assay. The results indicated that IGF2BP2 also binds to the 3'UTR of *Ucp1* mRNA (Figure 4H). The RNA-RNA interaction prediction also indicated that the most probable binding site between *Hilnc* and the *Ucp1* mRNA 3'UTR lies within the *Hilnc* 450-600 segment (Figure 4I), which aligns with our experimental findings. Furthermore, we performed molecular docking simulations between the IGF2BP2 structure, as provided by AlphaFold, and the three-dimensional structure of *Hilnc*. The green segment of the* Hilnc* molecule represents the region predicted to bind the *Ucp1* mRNA 3'UTR. Notably, this binding site is positioned in proximity to the IGF2BP2-binding region (Figure 4J), suggesting potential interactions among all three molecules.

The above results suggested that *Hilnc* may suppress *Ucp1* mRNA translation by facilitating IGF2BP2 binding to the 3'UTR of *Ucp1* mRNA.

### *Hilnc* Facilitates IGF2BP2-Mediated Suppression of *Ucp1* mRNA Translation

**Figure 5:**
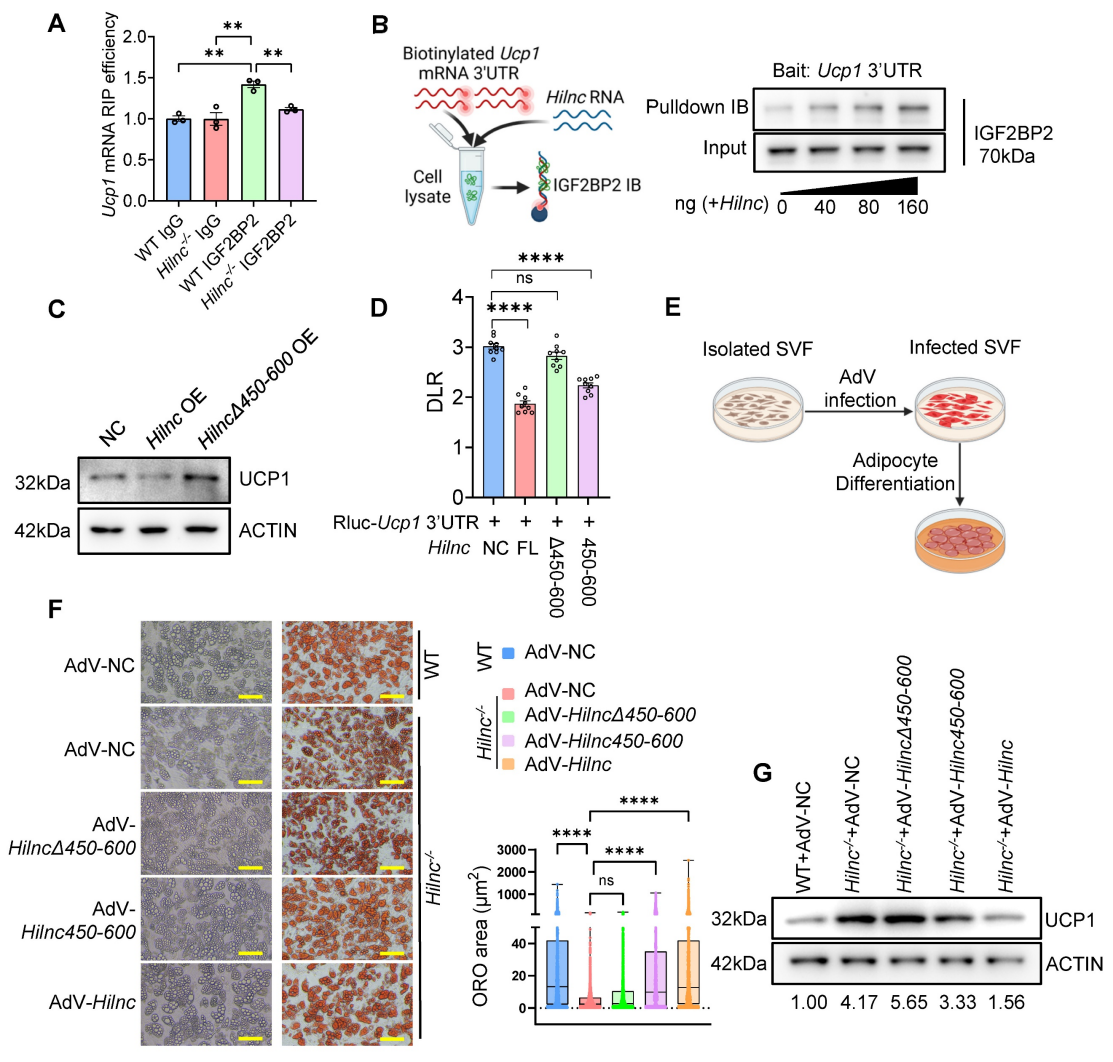
***Hilnc* facilitates IGF2BP2-mediated suppression of *Ucp1* mRNA. A)** IGF2BP2 RNA immunoprecipitation of *Ucp1* mRNA in WT and *Hilnc^-/-^* iWAT homogenates (N = 3 biological replicates). **B)** Left: Diagram of *Ucp1* mRNA 3'UTR pull down with addition of *Hilnc* RNA, and detection of IGF2BP2 by Western blot. Right: IGF2BP2 Western blot of *Ucp1* mRNA 3'UTR RNA pull-down with increasing concentrations of synthesized *Hilnc* RNA added. **C)** Western blot showing UCP1 expression in 3T3-L1 cells transfected with/without *Hilnc* FL or *Hilnc* Δ450-600. **D)** The Rluc-to-Fluc ratio (DLR) transfected psiCHECK2-Rluc-*Ucp1*-3'UTR into 3T3 cells, with an empty vector, FL *Hilnc*, *HilncΔ450-600* or *Hilnc450-600* co-transfection (N = 9 biological replicates). **E)** Diagram of SVF adenovirus (AdV) transfection and *in vitro* differentiation. **F)** Representative bright field images (left), oil-red O stain (middle) of infected and differentiated adipocytes, and quantification of lipid droplet area from Oil Red O Staining (right), showing lipid droplet sizes and lipid contents. Scale bar = 100 μm. **G)** Western blots of UCP1 expression in differentiated adipocytes infected with adenoviruses encoding *Hilnc* FL, *Hilnc* Δ450-600, or *Hilnc* 450-600. Bottom numbers indicate the grey scale ratio between UCP1 and ACTIN. Data are presented as mean ± SEM. One-way ANOVA with Tucky's multiple comparison correction (A, F) and Student's t-test (D) were used for statistical analysis. (ns: not significant, **: *p <* 0.01, ****: *p <* 0.0001).** See also Figure S6.**

Having identified the binding between *Hilnc*, IGF2BP2, and *Ucp1* mRNA, we sought to determine whether this interaction is responsible for the repression of *Ucp1* mRNA translation. RIP of IGF2BP2 from WT and *Hilnc^-/-^* iWAT revealed diminished binding between IGF2BP2 and *Ucp1* mRNA in the absence of *Hilnc* (Figure 5A). Furthermore, RNA pull-down experiments showed enhanced binding between IGF2BP2 and *Ucp1* mRNA with increasing concentrations of *Hilnc* (Figure 5B), demonstrating that *Hilnc* is essential for guiding IGF2BP2 to the 3'UTR of *Ucp1* mRNA.

After establishing the importance of *Hilnc* for the binding of IGF2BP2 to the 3'UTR of *Ucp1* mRNA, we next investigated whether *Hilnc* is necessary for translational suppression of *Ucp1* mRNA in the cellular context. To address this, we transfected 3T3-L1 cells, which express both IGF2BP2 and UCP1, with either FL *Hilnc* or a *Hilnc* variant lacking the 450-600nt region (*HilncΔ450-600*). The results showed that FL *Hilnc* strongly suppressed UCP1 translation, but *HilncΔ450-600*, which lacks the *Ucp1* mRNA-binding region, failed to repress *Ucp1* mRNA translation, confirming that binding to *Ucp1* mRNA is necessary for *Hilnc* to efficiently repress UCP1 translation (Figure 5C). To determine whether *Hilnc* repress *Ucp1* mRNA through its 3'UTR, we transfected psiCHECK2-Rluc-*Ucp1*-3'UTR (Figure S5C) into *Hilnc^-/-^* 3T3 cells, with co-transfecting of an empty vector, FL *Hilnc*, *HilncΔ450-600* or *Hilnc450-600.* The result showed that FL *Hilnc* strongly suppressed Rluc expression, while *HilncΔ450-600* had no significant effect. Notably, *Hilnc450-600* produced an intermediate but significant repression compared to the FL *Hilnc* transcript (Figure 5D). These findings suggest that *Hilnc* mediates *Ucp1* mRNA translational suppression through its 3'UTR, with the 400-600 region plays a critical role in this regulatory mechanism. To further investigate the impact of *Hilnc* on UCP1 translation in adipocytes, we employed the *in vitro* adipocyte differentiation system. SVF was isolated from *Hilnc^-/-^* mice and infected with adenoviruses (AdVs) encoding FL *Hilnc*, *HilncΔ450-600*, or *Hilnc450-600*, or negative control AdV (AdV-NC) prior to differentiation (Figure 5E, Figure S6A) to evaluate the specific effects of each *Hilnc* construct on adipocytes. Introduction of FL *Hilnc* into *Hilnc^-/-^* SVF did not significantly alter *Ucp1* mRNA levels (Figure S6B-C) but resulted in the formation of larger lipid droplets in differentiated adipocytes and decreased UCP1 protein levels, resembling those in WT differentiated adipocytes. SVF from *Hilnc^-/-^* mice infected with AdV-*HilncΔ450-600* formed smaller lipid droplets and failed to suppress UCP1 expression, similar to AdV-NC-infected *Hilnc^-/-^* SVF. In contrast, infection with AdV-*Hilnc450-600* in *Hilnc^-/-^* SVF resulted in smaller lipid droplets in differentiated adipocytes and a slight reduction in UCP1 levels compared to AdV-NC infected *Hilnc*^-/-^ SVF (Figure 5F-G). These findings suggest that the 450-600nt of *Hilnc* is critical for the repression of UCP1 translation.

In conclusion, our results demonstrate that *Hilnc* facilitates IGF2BP2-mediated translational suppression of *Ucp1* mRNA through interaction with its 3'UTR.

### *h*-*Hilnc*, the Human Functional Homolog of Mouse *Hilnc*, Operates through a Similar Mechanism in Human Adipose Tissues

**Figure 6:**
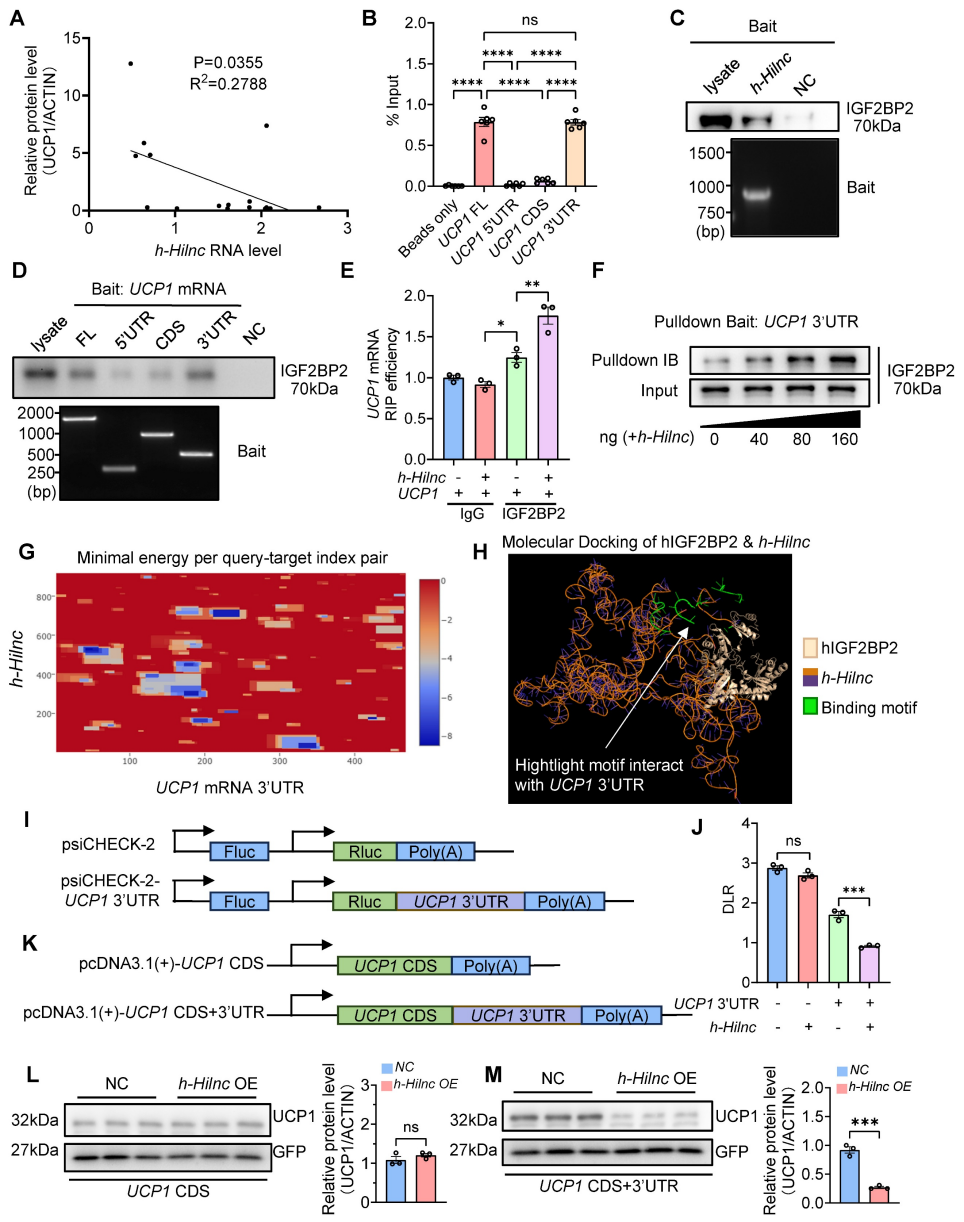
***h-Hilnc,* the human functional homolog of mouse *Hilnc*, may operate through a similar mechanism in human adipose tissues. A)** Correlation between each human adipose tissue biopsy's *h-Hilnc* RNA level and UCP1 protein level relative to ACTIN. (*p* = 0.0355, R^2^ = 0.2788, N = 16 samples). **B)** RT-qPCR of *in vitro* RNA-RNA binding assay between synthesized *h-Hilnc* RNA and biotin-conjugated fragments of *UCP1* mRNA (N = 3 technical replicates from 2 repeats). **C-D)** Western blots (top) showing RNA pull-down assays of IGF2BP2 from HEK293T cell lysate, using biotin-*h-Hilnc* (C) or *UCP1* mRNA segments (D) as baits (bottom), respectively. **E)** RT-qPCR of *UCP1* mRNA enriched by IGF2BP2 RIP from UCP1-transfected HEK293T cells, co-transfected with blank or *h-Hilnc* plasmid (N = 3 biological replicates). **F)** Detection of IGF2BP2 by Western blot of RNA pull-down assays using *UCP1* mRNA 3'UTR as bait, with increasing concentrations of synthesized *h-Hilnc* RNA added. **G)** *In silico* prediction of RNA-RNA interaction between *h-Hilnc* and *UCP1* mRNA 3'UTR (https://rna.informatik.uni-freiburg.de/IntaRNA/Result.jsp). **H)** Molecular docking of hIGF2BP2 & *h-Hilnc*. The green highlight indicates the segment on *h-Hilnc* RNA with the most favorable (lowest) binding free energy to the *UCP1* mRNA 3'UTR region. **I)** Construction strategy of PsiCHECK-2-*UCP1* 3'UTR. **J)** DLR of cells transfected with PsiCHECK-2 or PsiCHECK-2-*UCP1* 3'UTR, with or without co-transfection of *h-Hilnc* (N = 3 biological replicates). **K)** Construction strategy of pcDNA3.1(+)-*UCP1* plasmid, with or without the endogenous 3'UTR. **L)** Western blots analysis and quantification of UCP1 and co-transfected GFP protein levels in HEK293T cells transfected with pcDNA3.1(+)-*UCP1* CDS plasmid and with or without *h-Hilnc* (N = 3 technical replicates). **M)** Western blots analysis and quantification of UCP1 and co-transfected GFP protein levels in HEK293T cells transfected with pcDNA3.1(+)-*UCP1* CDS-*UCP1* 3'UTR plasmid and with or without *h-Hilnc* (N = 3 technical replicates). Data are presented as mean ± SEM. One-way ANOVA with Tucky's multiple comparison correction (D, G and J) was used for statistical analysis. (ns: not significant, *: *p <* 0.05, **: *p <* 0.01, ***: *p <* 0.001, ****: *p <* 0.0001).** See also Figure S7.**

Building on our findings in mice, we next investigated whether a similar mechanism might regulate UCP1 expression in humans. Specifically, we focused on *h-Hilnc*, the human functional homolog of mouse *Hilnc* identified in previous research 34. Using publicly available RNA-seq data, we identified *h-Hilnc* expression in human adipose tissue (Figure S7A), which was further confirmed by RT-PCR of human subcutaneous adipose tissue biopsy samples of various anatomical locations (Figure S7B). In addition, RT-qPCR of *h-Hilnc* showed that *h-Hilnc* levels vary among individual samples (Figure S7C), prompting us to examine whether such variation could correlate with the UCP1 protein level in human adipose tissues. Western blots showed that UCP1 protein level also varied among all samples (Figure S7D). We found that *h-Hilnc* level did not significantly correlate with *UCP1* mRNA level (Figure S7E), but negatively correlated with UCP1 protein levels significantly (Figure 6A), indicating that *h-Hilnc* could post-transcriptionally influence UCP1 protein level in human adipose tissues, similar to the function of *Hilnc* in mouse iWATs. We were then interested in whether the molecular interactions among *h-Hilnc*, IGF2BP2, and *UCP1* mRNA 3'UTR also existed. *In vitro* RNA binding assay confirmed the interaction between *h-Hilnc* and the 3'UTR of* UCP1* mRNA (Figure 6B). Additionally, both *h-Hilnc* and the 3'UTR of *UCP1* mRNA were able to bind to human IGF2BP2 (Figure 6C-D). Thus, the interactions among *h-Hilnc*, IGF2BP2, and *UCP1* mRNA in humans mirror those observed between their homologs in mice.

We next sought to determine whether these interactions could also suppress *UCP1* translation. In IGF2BP2 RIP experiments, we observed that *UCP1* mRNA was more efficiently enriched when *h-Hilnc* was present (Figure 6E), suggesting that similar to *Hilnc* in mice, *h-Hilnc* facilitates the binding of IGF2BP2 to the *UCP1* mRNA. Furthermore, RNA pull-down experiments showed that *h-Hilnc* facilitated binding between IGF2BP2 and the *UCP1* 3'UTR in a dose-dependent manner (Figure 6F). Based on the consistent mode of action revealed by the experimental results, we predicted the specific binding region between *h-Hilnc* and the* UCP1* mRNA 3'UTR. The prediction indicated a strong binding propensity between the 600-800 segment of *h-Hilnc* and the *UCP1* mRNA 3'UTR (Figure 6G). Molecular docking simulations between the IGF2BP2 structure (provided by AlphaFold) and the predicted three-dimensional structure of *h-Hilnc* showed that the predicted *h-Hilnc* region interacting with the *UCP1* mRNA 3'UTR is also positioned adjacent to the protein-binding site (Figure 6H), revealing a pattern similar to that observed in the mouse molecules. To confirm whether translational suppression of *h-Hilnc* and IGF2BP2 depends on the *UCP1* mRNA 3'UTR, we cloned the *UCP1* mRNA 3'UTR downstream of the Rluc CDS for dual-luciferase reporter assay (Figure 6I) and transfected the construct into HEK293T cells, with or without *h-Hilnc*. The results demonstrated that *h-Hilnc* suppressed Rluc translation exclusively when the *UCP1* mRNA 3'UTR was present (Figure 6J). To assess the impact of *h-Hilnc* on UCP1 protein levels, the 3'UTR of *UCP1* mRNA was cloned downstream of the *UCP1* coding sequence (CDS) in a mammalian expression plasmid (Figure 6K). HEK293T cells were co-transfected with plasmids encoding *h-Hilnc* and UCP1 with or without the 3'UTR, and a GFP-encoding plasmid for control of transfection efficiency. We found that *h-Hilnc* effectively suppressed UCP1 expression only when the 3'UTR was present (Figure 6L-M), confirming that *h-Hilnc* is able to suppress UCP1 mRNA translation via its 3'UTR.

In conclusion, our findings suggest that *h-Hilnc* suppressed UCP1 translation in human adipocytes through mechanisms similar to those observed in mouse adipocytes.

## Discussion

**Figure 7:**
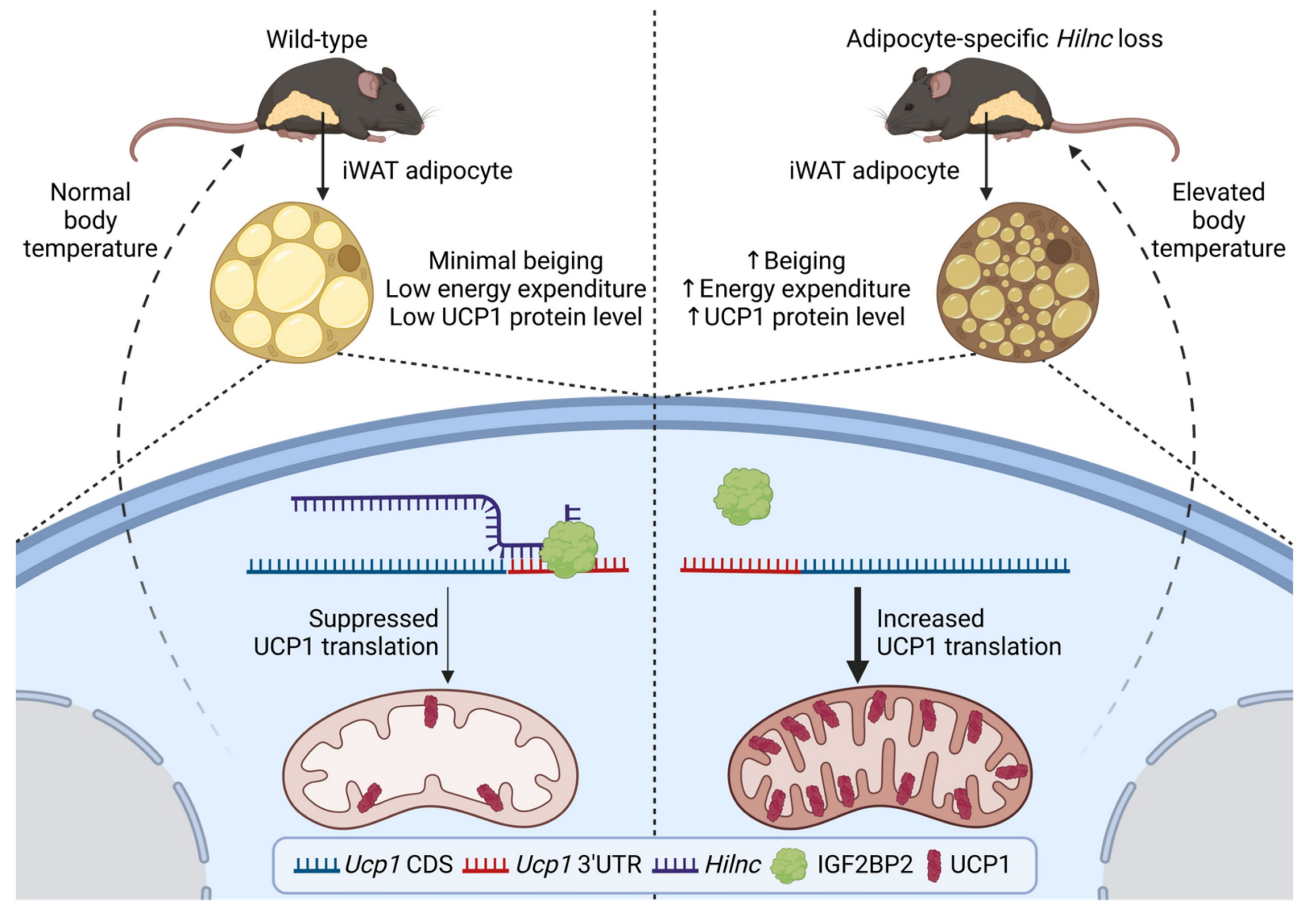
** Summary of the role of *Hilnc* in homeostatic thermogenesis through IGF2BP2-mediated translational repression of UCP1.** In the current study, we discovered that *Hilnc* plays an important role in regulation of UCP1 protein level in the beige adipocytes from iWATs of mice. Compared to the WT iWAT, iWATs from adipose-specific *Hilnc-*deficient mice exhibited elevated body temperature, increased beiging, elevated energy expenditure and increased UCP1 protein level. *Hilnc* was found to bind to the 3'UTR of *Ucp1* mRNA and facilitate recruitment of IGF2BP2 for the translational suppression of *Ucp1* mRNA.

The beiging of white adipose tissue has been proposed as an important mechanism for mammalian cold adaptation 7, 53, 54. This process is marked by increased thermogenesis and upregulation of the uncoupling protein UCP1 in white adipose tissue from certain depots in mice and humans 8, 10, 55, 56. The function of long non-coding RNAs (lncRNAs) in the beiging of adipocytes remains poorly understood. In this study, we demonstrate that *Hilnc*, a previously identified lipid metabolism-related lncRNA, plays a pivotal role in regulating UCP1 expression in beige adipocytes from iWATs. *Hilnc*-deficient iWATs underwent spontaneous UCP1 expression and increased thermogenesis, thereby contributing to the maintenance of homeostatic body temperature and cold adaptation in mice. The mechanism by which *Hilnc* exerts its effects was found to involve binding to the 3'UTR of *Ucp1* mRNA, while both RNA molecules bind to IGF2BP2. *Hilnc* facilitates the repression of *Ucp1* mRNA translation through the recruitment of IGF2BP2 to the *Ucp1* mRNA 3'UTR. The previously identified *Hilnc* functional homolog in humans, *h-Hilnc*, was found to regulate *Ucp1* mRNA translation in human adipocytes via a similar mechanism. In conclusion, the research proposed a model of how *Hilnc* contributes to homeostatic thermogenesis through IGF2BP2-mediated translational repression of UCP1 (Figure 7), providing additional insights into the gene regulation and function of adipocytes by lncRNA and highlighting the therapeutic potential of targeting lncRNAs for the regulation of body energy homeostasis.

The mitochondrial uncoupling protein UCP1 plays a central role in thermogenesis, enabling mammals to maintain body temperature in response to cold environments 57. UCP1 expression in thermogenic adipocytes, such as brown and beige adipocytes, is triggered by stimuli like cold exposure, β-adrenergic signaling, oxidative stress, and HFD 55, 58, 59. Expression of UCP1 is regulated at multiple levels. Recent studies have uncovered variable forms of the 3'UTR of *Ucp1* mRNA, suggesting its critical role as a regulatory target 60. However, the potential for lncRNAs to regulate UCP1 translation remains largely unexplored. Our discovery that *Hilnc* binds to the 3'UTR of *Ucp1* mRNA and recruits IGF2BP2 to suppress translation represents a significant advancement in understanding lncRNA-mediated post-transcriptional regulation. These findings also emphasize the importance of RNA-guided riboprotein complexes in directing and exerting regulatory functions on target mRNAs.

The interactions between different RNA species, such as mRNAs and non-coding RNAs, have garnered significant research interest due to their critical role in gene expression regulation. To date, four main archetypes of lncRNA-mediated gene regulation have been identified, involving interactions with macromolecules like DNA, RNA, and proteins 31, 61. Among these, the role of lncRNAs in regulating gene expression via DNA or chromatin interactions has been the most extensively studied 51, 62. Much of the existing research focuses on antisense mediated *in cis* regulation, which affects parent genes located on the opposite DNA strand 63. However, their interactions with mRNA, particularly *in trans*, remain less understood. Our discovery that *Hilnc* mediates *Ucp1* mRNA translation by recruiting IGF2BP2 highlights the underappreciated significance of lncRNA-mRNA interactions *in trans*. When the *Ucp1* mRNA-binding segment of *Hilnc* was abolished, IGF2BP2 failed to bind effectively to *Ucp1* mRNA, emphasizing the importance of RNA guidance in facilitating this interaction. Notably, this suppression mechanism did not involve *Ucp1* mRNA degradation (Figure 2D, J), which resembles miRNA-mediated translational suppression. miRNAs often target multiple genes simultaneously, raising the question of whether *Hilnc* or other lncRNAs may similarly regulate multiple genes *in trans*. If true, this could represent a broader and more systematic layer of gene expression control. Understanding these mechanisms may illuminate how lncRNAs like *Hilnc* orchestrate cellular functions and contribute to gene regulatory networks.

Despite their functional and marker similarities, beige and brown adipocytes are proposed to be distinct cell types originating from different lineages 7, 54. Brown adipocytes exhibit a partially myogenic transcriptional signature and are thought to have a closer relationship to the muscle lineage 64. In contrast, beige adipocytes have been proposed to arise either from beige-specific preadipocytes or through the trans-differentiation of white adipocytes 65-67. This suggests that the differential regulation of thermogenesis in brown and beige adipocytes may contribute to the complex thermogenic regulation observed at the whole-body level. Most existing studies that investigated the role of lncRNAs in the beiging of white adipocytes focused on the transcriptional regulation of thermogenic genes, and few studies, if any, explored the potential post-transcriptional regulation by lncRNAs. Our findings provide new insights from such perspective, revealing that as a lncRNA that is regulating UCP1 level post-transcriptionally, *Hilnc* is expressed in both eWAT and iWAT, but not in BAT. Furthermore, *Hilnc* was uniquely associated with UCP1 translation in beige adipocytes from iWAT, but not in eWAT or BAT. These results highlight the molecular differences between brown, beige, and white adipocytes, and suggest that distinct regulatory mechanisms govern UCP1 expression in brown and beige adipocytes despite their functional similarity. Considering that brown adipocytes were developmentally closer to the muscle lineage, and *Hilnc* was expressed in muscles 34 but not in brown adipocytes (Figure S1F), it raises an interesting speculation that the absence of *Hilnc* may be required for efficient expression of UCP1 in brown adipocytes during their differentiation.

In the investigation of the whole-body metabolism characteristics of mice by metabolic cages, we observed a shift in the energy metabolite preference of *Hilnc^AKO^* mice, but no significant differences in energy expenditure between *Hilnc^f/f^* and *Hilnc^AKO^* mice were observed. We reasoned that under homeostatic conditions, the mice were not experiencing significant metabolic stress, and the higher UCP1 protein level in the beige adipocytes of *Hilnc^AKO^* mice was unable to drive a significant difference in overall energy expenditure, and only manifested as a shift in relatively higher lipid consumption. The effect of *Hilnc* on overall energy expenditure was further illustrated when we subjected the mice to energy-demanding stress (NA injection; Figure 1L), showing that *Hilnc^AKO^* mice experience higher energy expenditure under stress, which is consistent with our hypothesis that *Hilnc* expression would affect overall energy expenditure of mice.

As our understanding of the functions of non-coding RNA has advanced in recent years, an increasing number of these molecules have been recognized as viable therapeutic targets for a variety of diseases, with many potential targeting strategies currently in clinical trials or under development 68. Metabolic disorders such as obesity have become increasingly prevalent worldwide and are closely linked to the incidence of other diseases, including diabetes, cardiovascular diseases, and musculoskeletal disorders, posing significant threats to overall human health and life expectancy 69-71. Given its role in energy dissipation, UCP1 has emerged as a promising therapeutic target for treating obesity and related metabolic disorders 56, 72-74. Previous studies have indicated that human visceral adipose tissues do not express UCP1 9, 75, rendering investigations of UCP1 expression in human adipose tissues difficult. Our study, which sampled subcutaneous adipose tissues, was able to detect UCP1 protein in the sampled tissues, and demonstrated that *h-Hilnc* levels negatively correlated with UCP1 protein levels in human adipose tissues, implying that *h-Hilnc* may be an intrinsic factor that regulates the function of differentiated human beige adipocytes, and overall homeostatic thermoregulation and energy expenditure of the human body. Specifically, by targeting and disrupting the *UCP1* mRNA-binding region, it may be possible to relieve the translational suppression of *UCP1* by IGF2BP2, leading to increased UCP1 expression and enhanced energy expenditure in human beige adipocytes, which could promote higher homeostatic body energy expenditure, potentially contributing to the fight against obesity.

In summary, our findings reveal the role of *Hilnc* in regulating beige adipocyte activity through the post-transcriptional regulation of UCP1 translation. This work highlights the complex regulation of adipocyte function and thermogenesis by lncRNAs at the post-transcriptional level, and provides insights into the potential therapeutic applications of modulating lncRNA function to target adipocyte activity.

## Supplementary Material

Supplementary figures and tables.

## Abbreviations

LncRNA: long non-coding RNA
UCP1: Uncoupling protein 1
SVF: stromal vascular fraction
iWAT: inguinal white adipose tissue
BAT: brown adipose tissue
eWAT: epidydimal white adipose tissues
TEM: Transmission electron microscopy
LC-MS/MS: Liquid Chromatography-Tandem Mass Spectrometry
RIP: RNA immunoprecipitation
RPA: RNase protection assay
3'UTR: 3' untranslated region
IGF2BP2: insulin-like growth factor 2 binding protein 2
HNRNPL: heterogeneous nuclear ribonucleoprotein L
AdV: adenovirus
*h-Hilnc*: human homolog of *Hilnc*
VO₂: oxygen consumption
VCO₂: carbon dioxide production
RER: respiratory exchange ratio
EE: energy expenditure
NA: Norepinephrine Bitartrate Monohydrate
SVF: Stromal Vascular Fraction
IBMX: Isobutyl-1-methylxanthine
T3: Triiodo-l-thyronine
MOI: multiplicity of infection
Fluc: firefly luciferase
Rluc: renilla luciferase
