# Supplementary material for: Hilnc-mediated UCP1 translation repression contributes to thermogenesis and energy expenditure
Source: Theranostics. 2026 Jan 1;16(7):3263–85. doi: 10.7150/thno.122688 (PMC12846010; doi:10.7150/thno.122688)
Supplement: Supplementary file 1 — Supplementary figures and tables. [file thnov16p3263s1.pdf]

Supplementary Materials for

Hilnc-mediated UCP1 translation repression contributes to thermogenesis and energy expenditure

Man Jiang[1, 2, #], Yu Li[1, #, *], Yiao Jiang[3, 4, #], Runze Wang[1], Jiayin Peng[1], Yuang Wang[1], Zhen Qu[1], Yi Chang[5, *], Zhao Zhang[3, 4, *], Yun Zhao[1,2,6, 7, *]

Affiliations

[1]Key Laboratory of Multi-Cell Systems, Shanghai Institute of Biochemistry and Cell Biology, Center for Excellence in Molecular Cell Science, Chinese Academy of Sciences, University of Chinese Academy of Sciences, Shanghai, China

[2]School of Life Science and Technology, ShanghaiTech University, Shanghai, China

[3]Center for the Genetics of Host Defense, University of Texas Southwestern Medical Center, Dallas, TX, USA

[4]Division of Endocrinology, Department of Internal Medicine, University of Texas Southwestern Medical Center, Dallas, TX, USA

[5]Department of Medical Aesthetic, Yangpu Hospital, Tongji University School of Medicine, Shanghai, China

[6]School of Life Science, Hangzhou Institute for Advanced Study, University of Chinese Academy of Sciences, Hangzhou, China

[7]Lead contact

[#]These authors contributed equally

[*]Correspondence: liyu@sibcb.ac.cn (Y.L.), chani_1213@163.com (Y.C), zhao.zhang@UTSouthwestern.edu (Z.Z.), yunzhao@sibcb.ac.cn (Z.Y.)

This supplementary view includes:

Figure S1 to S7
Table S1 to S4

Figure S1

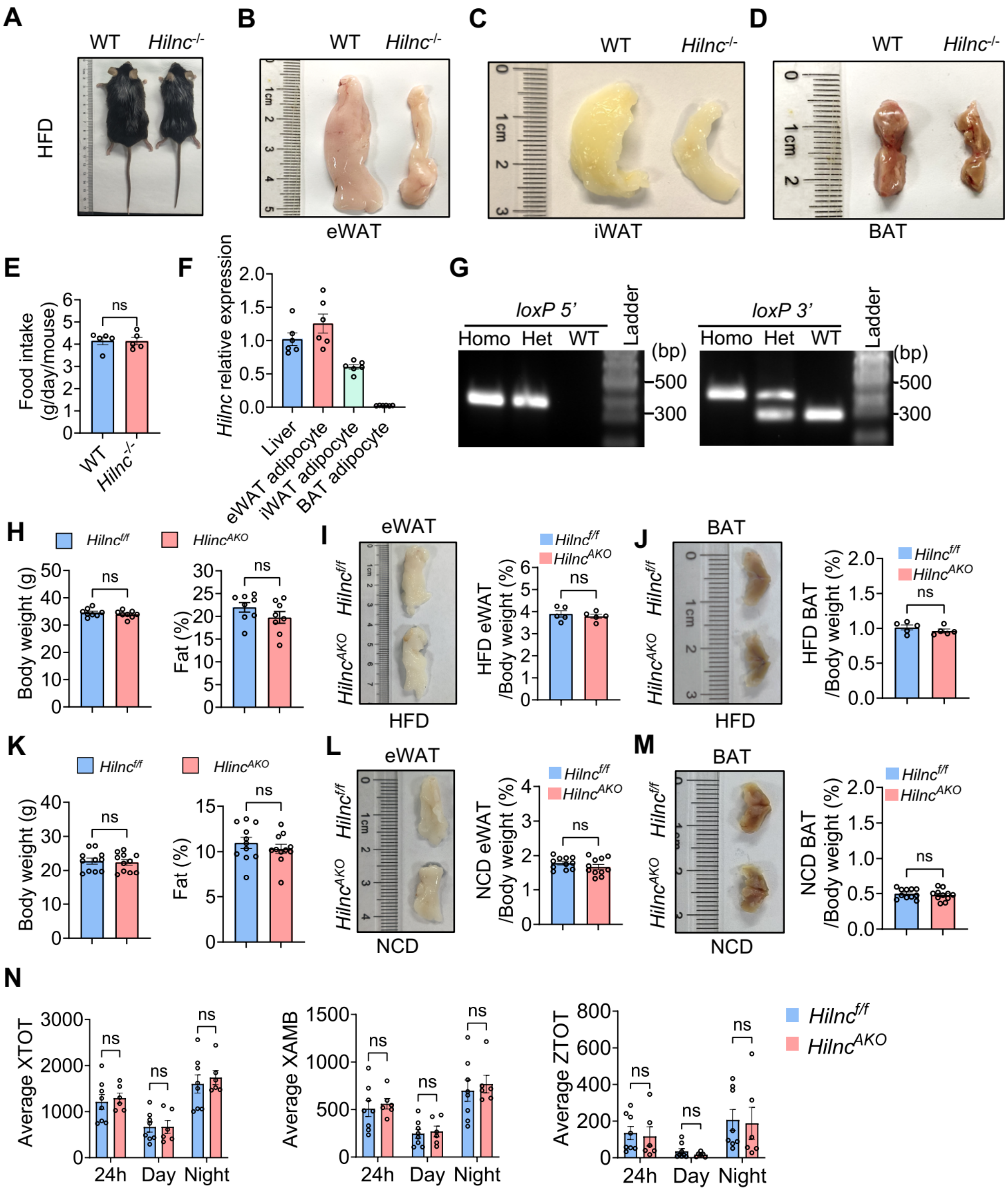
A
WT
Hilnc-/-
HFD
B
eWAT
C
iWAT
D
BAT
E
Food intake (g/day/mouse)
ns
F
Hilnc relative expression
Liver
eWAT adipocyte
iWAT adipocyte
BAT adipocyte
G
loxP 5'
loxP 3'
Homo
Het
Ladder
(bp)
500
300
H
Hilnc f/f
Hilnc AKO
Body weight (g)
Fat (%)
I
HFD
HFD eWAT /Body weight (%)
J
HFD BAT /Body weight (%)
K
L
NCD
NCD eWAT /Body weight (%)
M
NCD BAT /Body weight (%)
N
Average XTOT
Average XAMB
Average ZTOT
24h
Day
Night

Figure S1. *Hilnc* expression and associated phenotypes vary across adipose tissues. Related to Figure 1.

A-D) Comparison of body size (A), eWAT (B), iWAT (C), and BAT (D) morphology between HFD-fed WT and *Hilnc*$^{-/-}$ mice.

E) Comparison of food intake between HFD-fed WT and *Hilnc*$^{-/-}$ mice (N = 5 mice for each strain).

F) RT-qPCR of *Hilnc* RNA levels in various adipocytes, relative to *Hilnc* level in the liver (N = 3 technical replicates of 2 mice from each strain).

G) Genotyping strategy of *Hilnc*$^{f/f}$ mice.

H) Body weight and body fat percentage of HFD-fed *Hilnc*$^{f/f}$ and *Hilnc*^{AKO} mice (N = 8 mice for each strain).

I) Representative morphology of eWAT and the mass of eWAT as a percentage of body weight of HFD-fed *Hilnc*$^{f/f}$ and *Hilnc*^{AKO} mice (N = 5 mice for each strain).

J) Representative morphology of BAT and the mass of BAT as a percentage of body weight of HFD-fed *Hilnc*$^{f/f}$ and *Hilnc*^{AKO} mice (N = 5 mice for each strain).

K) Body weight and body fat percentage of NCD-fed *Hilnc*$^{f/f}$ and *Hilnc*^{AKO} mice (N = 11 mice for each strain).

L) Representative morphology of eWAT and the mass of eWAT as a percentage of body weight of NCD-fed *Hilnc*$^{f/f}$ and *Hilnc*^{AKO} mice (N = 11 mice for each strain).

M) Representative morphology of eWAT and the mass of eWAT as a percentage of body weight of NCD-fed *Hilnc*$^{f/f}$ and *Hilnc*^{AKO} mice (N = 11 mice for each strain).

N) Averages of total XY movement (XTOT), ambulatory XY movement (XAMB), and total Z movement (ZTOT) between NCD-fed *Hilnc*$^{f/f}$ and *Hilnc*^{AKO} mice (N = 8 mice for *Hilnc*$^{f/f}$ and N = 6 mice for *Hilnc*^{AKO}).

Data are presented as mean ± SEM. Student's t-tests were used for statistical analysis, with Holm-Šídák's multiple comparison correction applied (N). (ns: not significant)

Figure S2

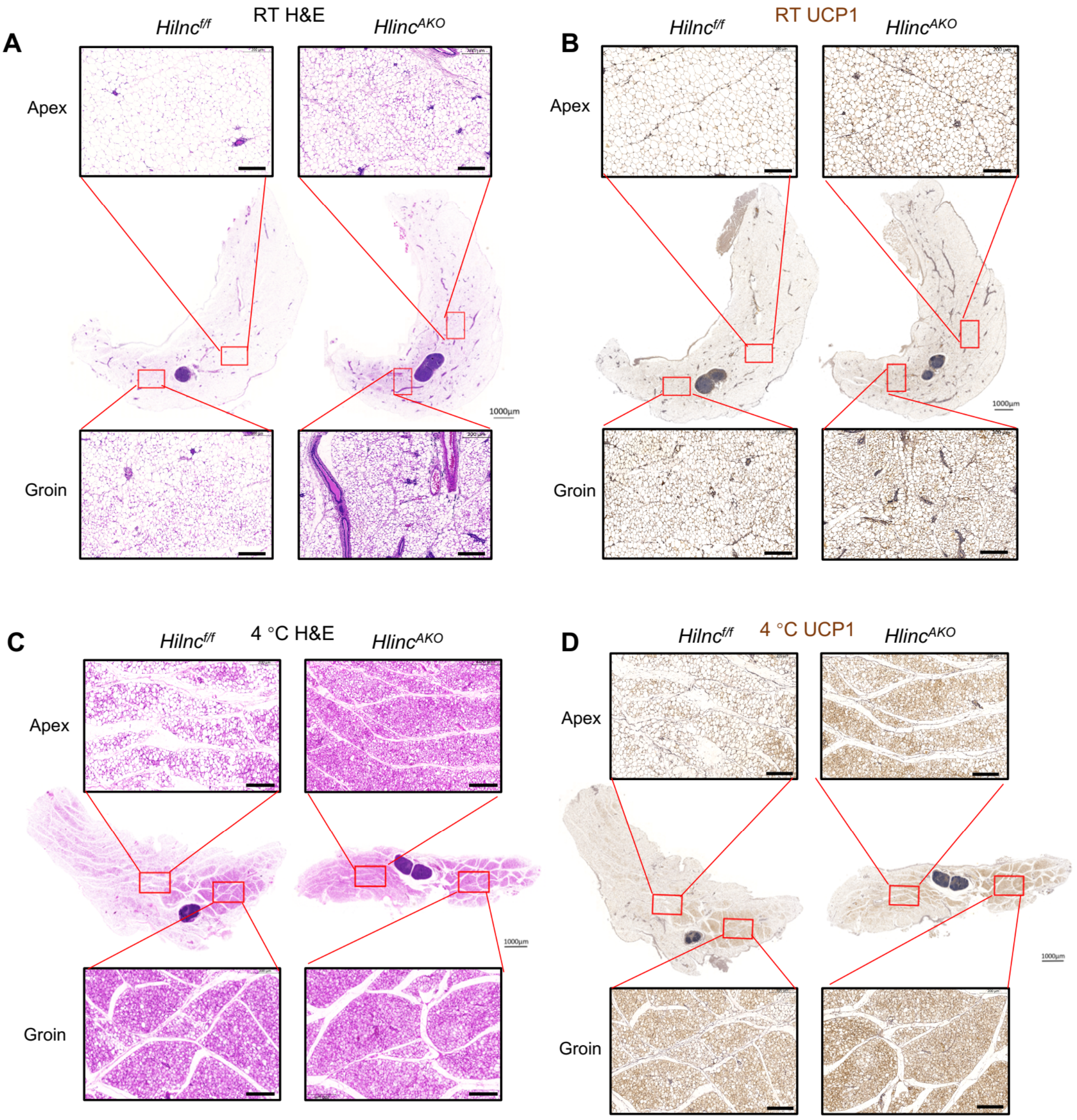

A
RT H&E
Hilnc f/f
Hlinc AKO
Apex
Groin
1000μm
B
RT UCP1
Hilnc f/f
Hlinc AKO
Apex
Groin
1000μm
C
4 °C H&E
Hilnc f/f
Hlinc AKO
Apex
Groin
1000μm
D
4 °C UCP1
Hilnc f/f
Hlinc AKO
Apex
Groin
1000μm

Figure S2. Group differences across environmental temperatures. Related to Figure 2.

A) Global H&E staining of iWAT from *Hilnc*$^{f/f}$ and *Hilnc*^{AKO} mice under RT conditions (middle, scale bar = 1000 µm), with magnified views of the apex (top) and groin (bottom) regions (Scale bar = 200 µm).

B) Global UCP1 immunohistochemistry (IHC) of iWAT from *Hilnc*$^{f/f}$ and *Hilnc*^{AKO} mice under RT conditions (middle, scale bar = 1000 µm), with magnified views of the apex (top) and groin (bottom) regions (Scale bar = 200 µm).

C) Global H&E staining of iWAT from *Hilnc*$^{f/f}$ and *Hilnc*^{AKO} mice under 4 °C conditions (middle, scale bar = 1000 µm), with magnified views of the apex (top) and groin (bottom) regions (Scale bar = 200 µm).

D) Global UCP1 immunohistochemistry (IHC) of iWAT from *Hilnc*$^{f/f}$ and *Hilnc*^{AKO} mice under 4 °C conditions (middle, scale bar = 1000 µm), with magnified views of the apex (top) and groin (bottom) regions (Scale bar = 200 µm).

Figure S3

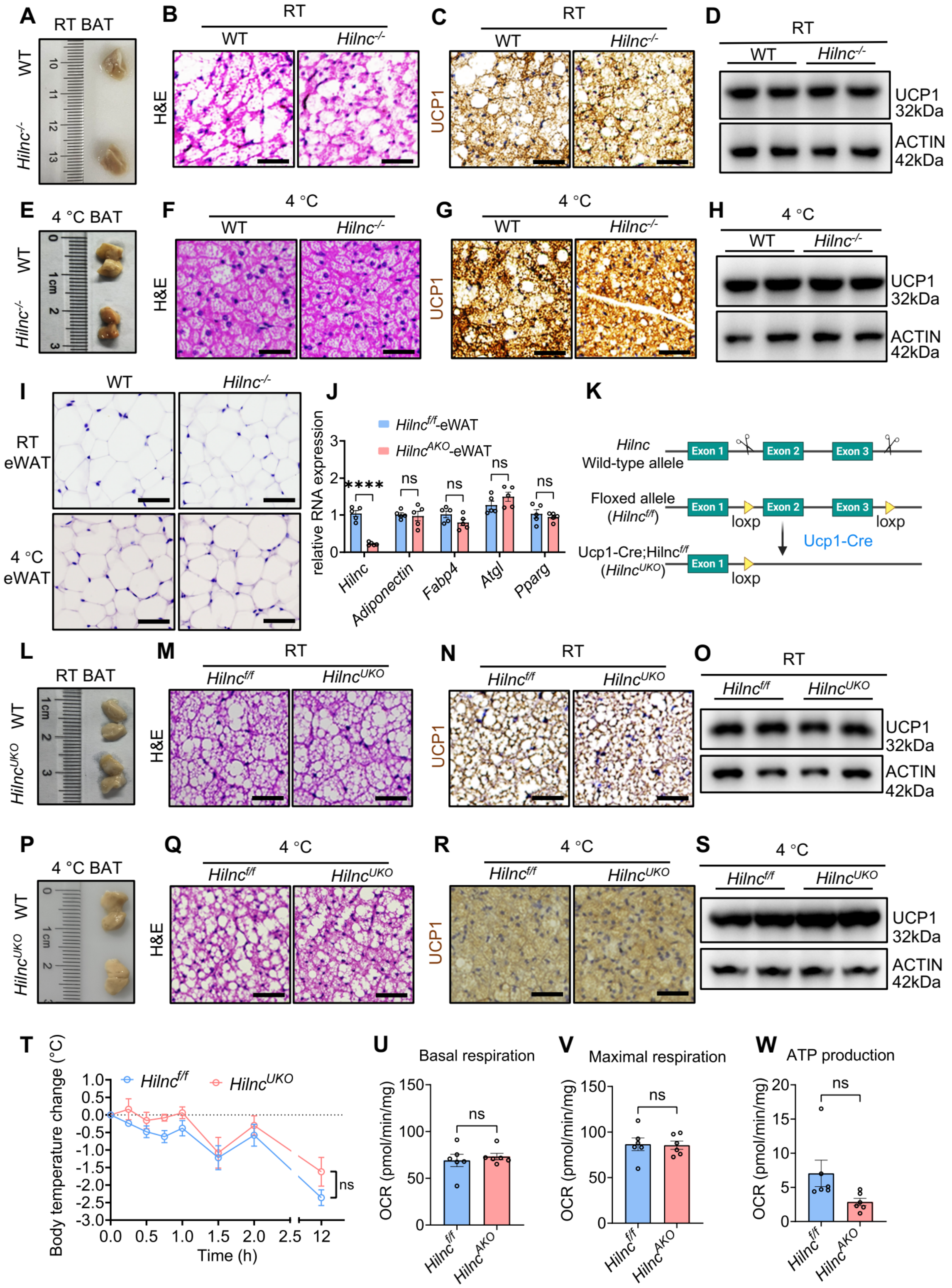

A RT BAT
WT
Hilnc-/-
B RT
WT Hilnc-/-
H&E
C RT
UCP1
D RT
UCP1 32kDa
ACTIN 42kDa
E 4 °C BAT
F 4 °C
G 4 °C
H 4 °C
I WT Hilnc-/-
RT eWAT
4 °C eWAT
J
Hilnc f/f-eWAT
Hilnc AKO-eWAT
relative RNA expression
Hilnc Adiponectin Fabp4 Atgl Pparg
ns
K
Hilnc Wild-type allele
Exon 1 Exon 2 Exon 3
Floxed allele (Hilnc f/f)
loxp
Ucp1-Cre
Ucp1-Cre;Hilnc f/f (Hilnc UKO)
L RT BAT
Hilnc UKO
M RT
Hilnc f/f Hilnc UKO
N RT
O RT
P 4 °C BAT
Q 4 °C
R 4 °C
S 4 °C
T
Body temperature change (°C)
Time (h)
U Basal respiration
V Maximal respiration
W ATP production
OCR (pmol/min/mg)
Hilnc f/f Hilnc AKO

Figure S3. No difference in BAT & WAT morphology and UCP1 expression was observed in *Hilnc*-deficient mouse models. Related to Figure 2.

A-D) Comparison of size (A), H&E stain (B), UCP1 IHC (C), and UCP1 Western Blot (D) in BAT from WT and *Hilnc*$^{-/-}$ mice at RT. Scale bar = 50 µm for B) and C).

E-H) Comparison of size (E), H&E stain (F), UCP1 IHC (G), and UCP1 Western blot (H) in BAT from cold-acclimated WT and *Hilnc*$^{-/-}$ mice (N = 2 mice for each strain). Scale bar = 50 µm for F) and G).

I) H&E stains of eWATS from RT or cold-acclimated, WT and *Hilnc*$^{-/-}$ mice. Scale bar = 50 µm.

J) qPCR analysis of multiple genes in the lipid metabolic pathway in mature adipocytes from eWAT of *Hilnc*$^{f/f}$ and *Hilnc*^{UKO} mice following 4 °C acclimation (N = 5 mice for each strain).

K) Strategy of generating *Ucp1-Cre; Hilnc*$^{f/f}$ (*Hilnc*^{UKO}) mice.

L-O) Comparison of size (K), H&E stain (L), UCP1 IHC (M), and UCP1 Western blot (N) in BAT from *Hilnc*$^{f/f}$ and *Hilnc*^{UKO} mice at RT. Scale bar = 50 µm for L) and M).

P-S) Comparison of size (O), H&E stain (P), UCP1 IHC (Q), and UCP1 Western blot (R) in BAT from cold-acclimated *Hilnc*$^{f/f}$ and *Hilnc*^{UKO} mice (N = 2 mice for each strain). Scale bar = 50 µm for P) and Q).

T) Average changes in rectal temperature of cold-acclimated *Hilnc*$^{f/f}$ and *Hilnc*^{UKO} mice upon re-exposure to cold (N = 5 mice for each strain).

U-W) Basal respiration (U), maximal respiration (V) and ATP production (W) in cold-acclimated *Hilnc*$^{f/f}$ and *Hilnc*^{AKO} iWATs (N = 6 biological replicates).

Data are presented as mean ± SEM. Student's t-tests were used for statistical analysis (J, T, U, V and W). (ns: not significant, ****: $p < 0.0001$)

Figure S4

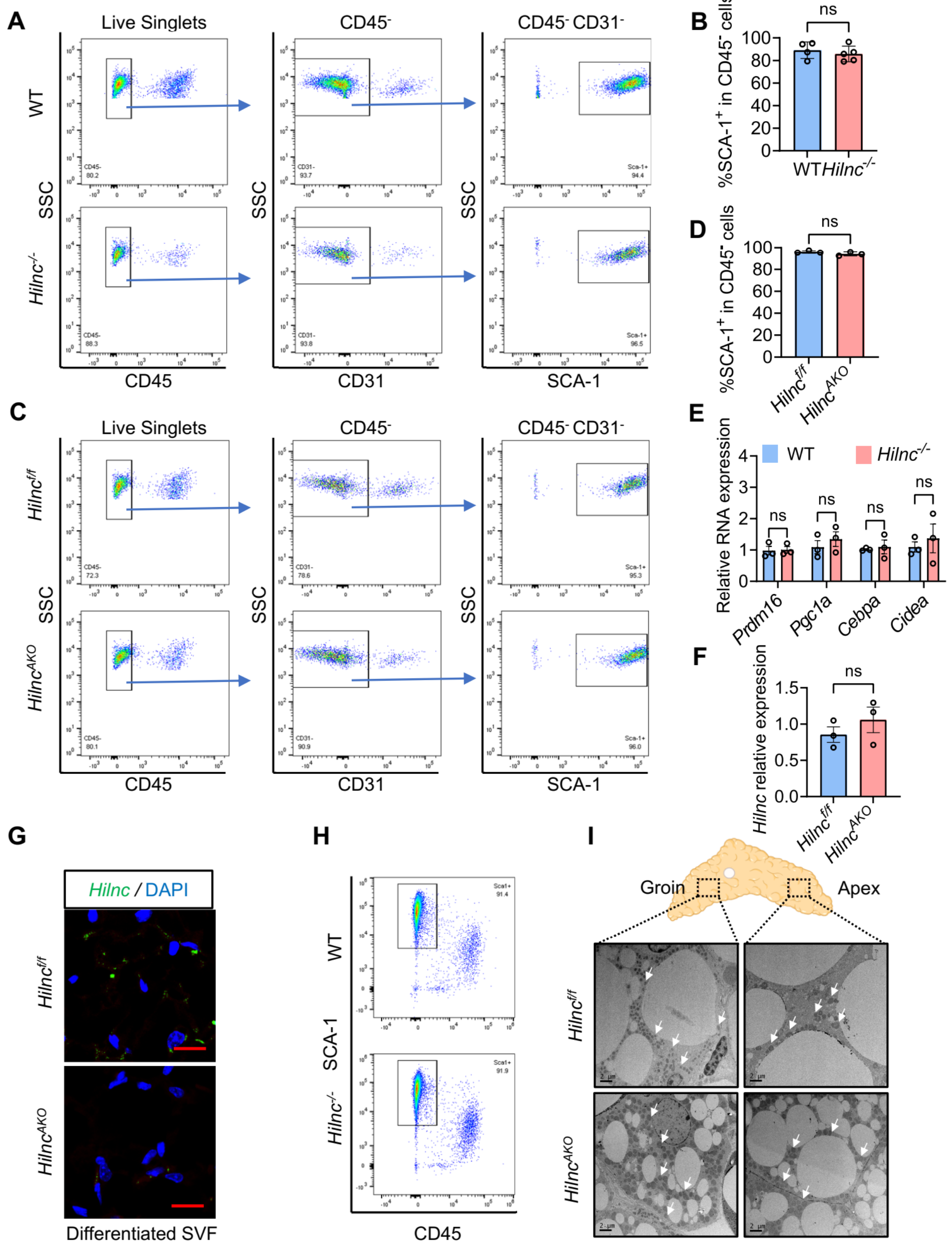

A
Live Singlets
CD45-
CD45- CD31-
WT
Hilnc-/-
SSC
CD45
CD31
SCA-1
B
ns
%SCA-1+ in CD45- cells
WT
Hilnc-/-
D
Hilncf/f
HilncAKO
E
WT
Hilnc-/-
Relative RNA expression
Prdm16
Pgc1a
Cebpa
Cidea
C
F
Hilnc relative expression
G
Hilnc / DAPI
Differentiated SVF
H
SCA-1
I
Groin
Apex

Figure S4. *Hilnc* deficiency in SVF does not contribute to the observed changes in iWAT beiging. Related to Figure 3.

A-B) Representative flow cytometry stains (A) and quantifications of frequencies (B) of $CD45^-CD31^-SCA\text{-}1^+$ preadipocytes among SVF cells from WT and *Hilnc*$^{-/-}$ iWAT (N = 4 mice for WT and N = 5 mice for *Hilnc*$^{-/-}$).

C-D) Representative flow cytometry stains (C) and quantifications of frequencies (D) of $CD45^-CD31^-SCA\text{-}1^+$ preadipocytes among SVF cells from *Hilnc*$^{f/f}$ and *Hilnc*^{AKO} iWAT (N = 3 mice for each strain).

E) RT-qPCR analysis of expression levels of key regulators of adipogenesis and beiging in SVFs from WT and *Hilnc*$^{-/-}$ iWAT (N = 3 technical replicates from 2-3 biological replicates).

F) RT-qPCR of *Hilnc* in plated adherent SVFs from *Hilnc*$^{f/f}$ and *Hilnc*^{AKO} iWAT (N = 3 mice for each strain).

G) *In situ* fluorescent hybridization (FISH) of *Hilnc* in *Hilnc*$^{f/f}$ and *Hilnc*^{AKO} *in vitro* differentiated adipocytes. Scale bar = 10 µm.

H) Representative flow cytometry stains of $CD45^-SCA\text{-}1^+$ preadipocytes among plated adherent SVF cells from WT and *Hilnc*$^{-/-}$ iWAT.

I) Representative TEM images of the groin and apex ends of iWAT from cold-acclimated *Hilnc*$^{f/f}$ and *Hilnc*^{AKO} mice, showing size and density of mitochondria within adipocytes. Arrows indicate locations of mitochondria. Scale bar = 2 µm.

Data are presented as mean ± SEM. Student's t-tests were used for statistical analysis (B, D, E, and F), with Holm-Šídák's multiple comparison correction applied (E). (ns: not significant)

Figure S5

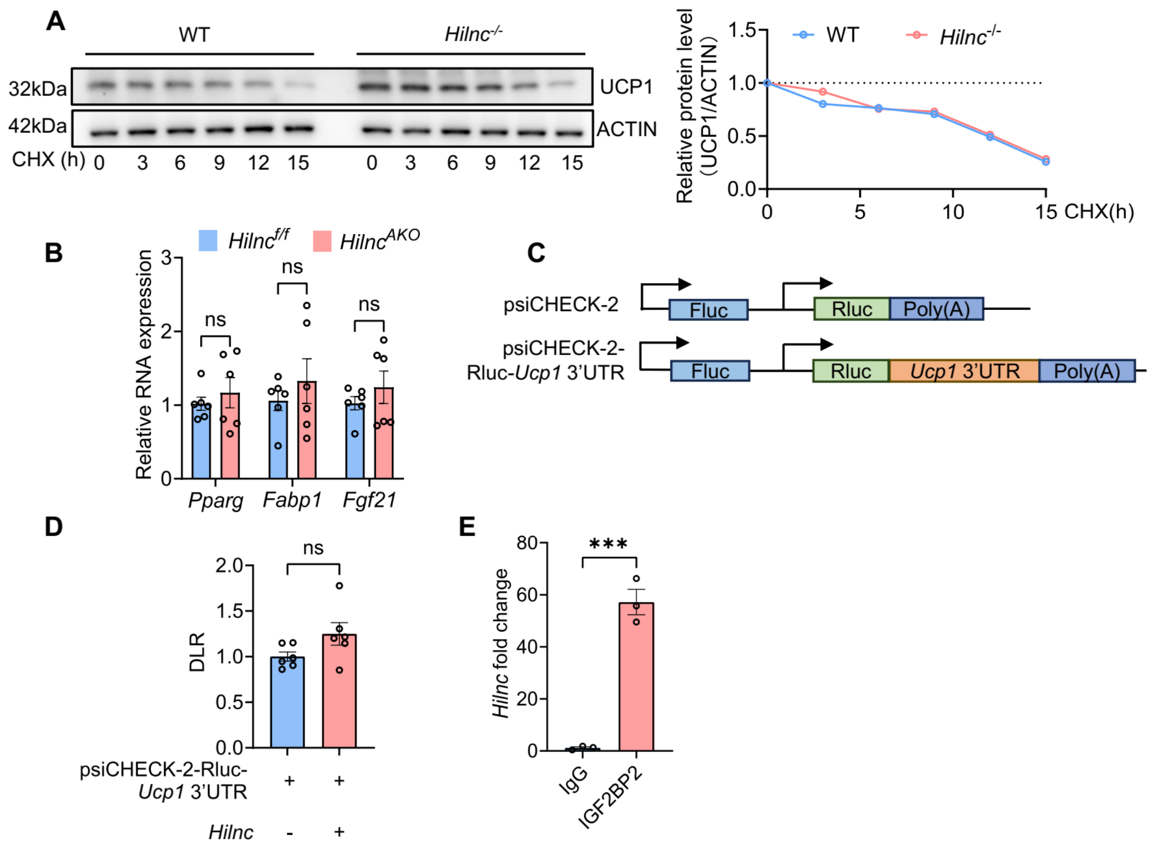
A
WT
Hilnc-/-
32kDa
42kDa
UCP1
ACTIN
CHX (h) 0 3 6 9 12 15 0 3 6 9 12 15
Relative protein level (UCP1/ACTIN)
CHX(h)
B
Hilncf/f
HilncAKO
Relative RNA expression
ns
Pparg
Fabp1
Fgf21
C
psiCHECK-2
psiCHECK-2-Rluc-Ucp1 3'UTR
Fluc
Rluc
Poly(A)
Ucp1 3'UTR
D
DLR
psiCHECK-2-Rluc-Ucp1 3'UTR
Hilnc
E
***
Hilnc fold change
IgG
IGF2BP2

Figure S5. No impact of *Hilnc* on UCP1 transcription, post-translational stability, and PPARγ signaling in adipose tissue. Related to Figure 4.

A) Western blots (left) and relative quantifications (right) of CHX-treated, *in vitro* differentiated adipocytes from SVFs of WT and $Hilnc^{-/-}$ mice.

B) RT-qPCR analysis of expression of *Pparg*, *Fabp1*, and *Fgf21* in cold-acclimated $Hilnc^{f/f}$ vs $Hilnc^{AKO}$ iWAT adipocytes (N = 3 technical replicates from 2 biological replicates).

C) Construction strategy of PsiCHECK-2-*Ucp1* 3'UTR plasmid.

D) The Rluc-to-Fluc ratio (DLR) of *in vitro* transcription and translation of PsiCHECK-2-*Ucp1* 3'UTR plasmid, with blank or *Hilnc*-expressing plasmid (N = 3 technical replicates from 2 biological replicates).

E) RT-qPCR of *Hilnc* enrichment by RNA immunoprecipitation of IGF2BP2 (N = 3 biological replicates).

Data are presented as mean ± SEM. Student's t-tests were used for statistical analysis (B, D and E), with Holm-Šídák's multiple comparison correction applied (B). (ns: not significant, ***: $p < 0.001$)

Figure S6

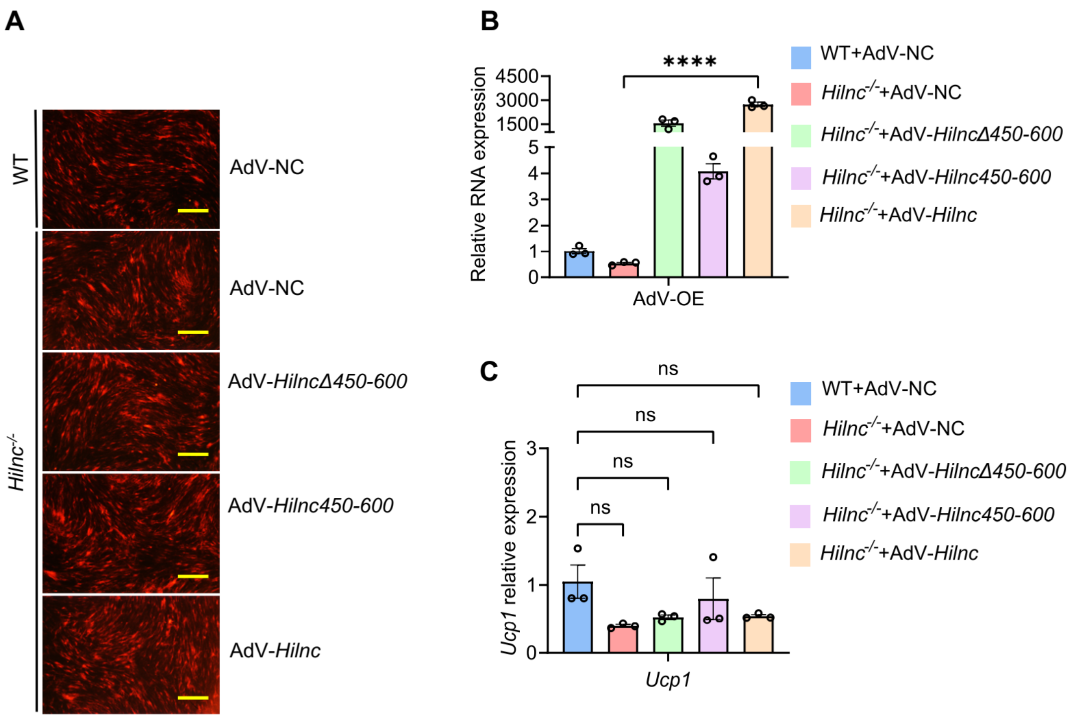
A
WT
AdV-NC
Hilnc-/-
AdV-NC
AdV-HilncΔ450-600
AdV-Hilnc450-600
AdV-Hilnc
B
Relative RNA expression
4500
3000
1500
5
4
3
2
1
0
****
AdV-OE
WT+AdV-NC
Hilnc-/-+AdV-NC
Hilnc-/-+AdV-HilncΔ450-600
Hilnc-/-+AdV-Hilnc450-600
Hilnc-/-+AdV-Hilnc
C
ns
ns
ns
ns
Ucp1 relative expression
3
2
1
0
Ucp1
WT+AdV-NC
Hilnc-/-+AdV-NC
Hilnc-/-+AdV-HilncΔ450-600
Hilnc-/-+AdV-Hilnc450-600
Hilnc-/-+AdV-Hilnc

Figure S6. Adenovirus-infected SVFs retain ability to differentiate into mature adipocytes. Related to Figure 5.

A) AdV infection efficiency evaluated by AdV-encoded mCherry expression in infected SVFs. Scale bar = 500 µm.

B) RT-qPCR of the expression of *Hilnc* constructs in infected and differentiated SVFs (N = 3 biological replicates).

C) RT-qPCR of *Ucp1* mRNA level in infected and differentiated SVFs (N = 3 biological replicates).

Data are presented as mean ± SEM. One-way ANOVA with Dunnett's multiple comparison correction (B and C) was used for statistical analysis. (ns: not significant, ****: $p < 0.0001$)

Figure S7

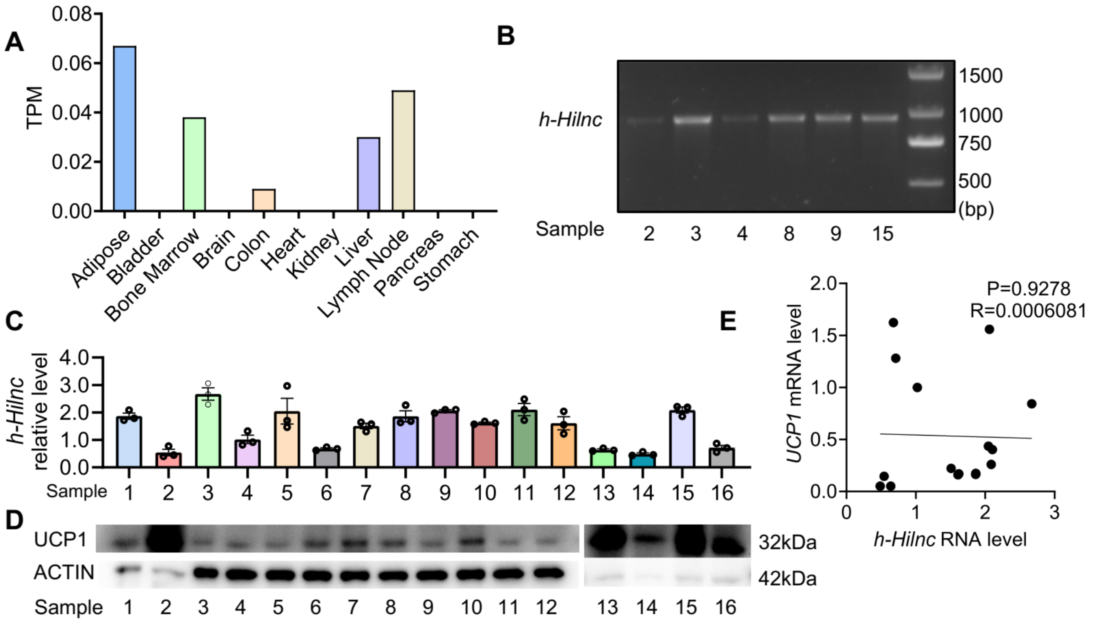

A
TPM
0.08
0.06
0.04
0.02
0.00
Adipose
Bladder
Bone Marrow
Brain
Colon
Heart
Kidney
Liver
Lymph Node
Pancreas
Stomach
B
h-Hilnc
1500
1000
750
500
(bp)
Sample 2 3 4 8 9 15
C
h-Hilnc relative level
4.0
3.0
2.0
1.0
0.0
Sample 1 2 3 4 5 6 7 8 9 10 11 12 13 14 15 16
D
UCP1
ACTIN
32kDa
42kDa
Sample 1 2 3 4 5 6 7 8 9 10 11 12 13 14 15 16
E
P=0.9278
R=0.0006081
UCP1 mRNA level
2.0
1.5
1.0
0.5
0.0
0 1 2 3
h-Hilnc RNA level

Figure S7. *h-Hilnc* is identified in human adipose tissue. Related to Figure 6.

A) Data retrieved from LncExpDB (https://ngdc.cncb.ac.cn/lncexpdb/) showing expression of HSALNG0010093, the gene locus from which *h-Hilnc* (ENST00000417084.1) is transcribed, in various tissues.

B) RT-PCR of *h-Hilnc* (ENST00000417084.1) from subcutaneous human adipose tissue RNAs. The corresponding patient sample numbers are labelled below each sample. (N = 6 samples).

C) RT-qPCR of relative *h-Hilnc* RNA level in human subcutaneous adipose tissue biopsies. The corresponding patient sample numbers are labelled below each sample. (N = 16 samples)

D) Western blot of UCP1 and ACTIN protein levels in human subcutaneous adipose tissue biopsies. The corresponding patient sample numbers are labelled below each sample. (N = 16 samples)

E) Correlation between each human adipose tissue biopsy's *h-Hilnc* RNA level and *UCP1* mRNA level. (p = 0.9278, R^2 = 0.0006081, N = 16 samples)

Data are presented as mean ± SEM.

Table S1.

List of top 30 candidate protein groups of *Hilnc*-bound or *Ucp1* mRNA-bound proteins from the LC-MS/MS analysis, ranked by the respective absolute intensity of the detected RNA-bound protein groups.

***Hilnc*-bound top 30 candidates**				***Ucp1* mRNA-bound top 30 candidates**			
**Gene Names**	**Intensity Ctrl**	**Intensity *Hilnc***	**Relative Intensity**	**Gene Names**	**Intensity Ctrl**	**Intensity *Ucp1* mRNA**	**Relative Intensity**
Hnrnpk	1.62E+10	1.82E+11	11.2	Actbl2	9.13E+07	3.91E+10	427.9
Ptbp1	9.08E+09	1.56E+11	17.2	Eif2ak2	8.81E+08	1.73E+10	19.7
Hdlbp	1.69E+10	8.69E+10	5.1	Ybx1	3.91E+09	1.16E+10	3.0
Pcbp2	2.60E+09	5.95E+10	22.8	Igf2bp3	5.18E+09	1.04E+10	2.0
**Igf2bp2[a]**	**4.57E+09**	**4.54E+10**	**9.9**	**Hnrnpl[a]**	**1.60E+09**	**9.37E+09**	**5.8**
**Hnrnpl[a]**	**2.04E+10**	**4.29E+10**	**2.1**	Pccb	3.64E+09	8.23E+09	2.3
Matr3	1.40E+09	2.73E+10	19.5	**Igf2bp2[a]**	**2.92E+09**	**6.72E+09**	**2.3**
Khsrp	4.22E+09	1.94E+10	4.6	Luc7l2	3.02E+09	6.12E+09	2.0
U2af2	5.81E+09	1.55E+10	2.7	Ybx3	1.05E+09	4.55E+09	4.3
Khdrbs1	4.01E+09	1.39E+10	3.5	Syncrip	1.93E+09	4.53E+09	2.3
Rtcb	5.79E+09	1.33E+10	2.3	Hnrnpa0	1.30E+09	2.90E+09	2.2
Fubp3	1.88E+09	1.27E+10	6.8	Hmgb1	8.04E+08	1.95E+09	2.4
Sf1	3.41E+09	1.15E+10	3.4	Hnrnpr	3.42E+08	1.89E+09	5.5
Pcbp1	5.91E+08	1.14E+10	19.2	Ptn	1.20E+08	1.80E+09	15.1
Rrp36	0.00E+00	1.09E+10	#DIV/0!	Zfr	3.40E+08	1.74E+09	5.1
Hnrnpd	2.86E+09	9.27E+09	3.2	Lsg1	6.79E+08	1.63E+09	2.4
U2af1	3.69E+09	9.25E+09	2.5	Arpc5	3.86E+08	1.45E+09	3.7
Sf3b1	4.74E+09	9.10E+09	1.9	Atxn2l	5.22E+08	1.25E+09	2.4
Ptbp3	3.25E+08	8.49E+09	26.1	Hnrnpf	5.54E+08	1.24E+09	2.2
Rpl22	2.56E+08	7.96E+09	31.1	2010002M12Rik;Gm14446	2.07E+08	1.16E+09	5.6
Zc3h4	3.48E+08	6.80E+09	19.5	Helz2	1.48E+08	1.13E+09	7.6
Pabpn1;Gm20521	6.88E+08	6.67E+09	9.7	Syne3	0.00E+00	1.12E+09	#DIV/0!
Fubp1	2.12E+09	6.61E+09	3.1	Snrnp40	4.93E+08	1.03E+09	2.1
Hnrnpll	1.29E+09	6.16E+09	4.8	Zfp948;Zfp708;…	0.00E+00	1.01E+09	#DIV/0!
Plec	8.08E+08	5.44E+09	6.7	Srsf9	3.79E+08	9.88E+08	2.6
Puf60	1.61E+09	5.39E+09	3.4	Rplp1	1.19E+08	9.68E+08	8.1

Ago2	8.17E+08	5.38E+09	6.6	Ddx56	4.09E+08	9.39E+08	2.3
Ptbp2	0.00E+00	5.33E+09	#DIV/0!	Nufip2	4.17E+08	8.42E+08	2.0
Raver1	6.29E+08	4.39E+09	7.0	Cacna1a	0.00E+00	8.39E+08	#DIV/0!
Csde1	9.76E+08	4.36E+09	4.5	Cpsf2	3.93E+08	8.23E+08	2.1

[a] Bold gene entries denote genes found in both *Hilnc*-bound top 30 candidates (left) and *Ucp1* mRNA-bound top 30 candidates (right) lists.

Table S2.

List of protein groups from LC-MS/MS analysis that are enriched in both *Ucp1* mRNA and *Hilnc* RNA pull-down experiments.

**Enrichment Ratio > 2**				
Ago2	Csde1	Fubp3	Mrpl9	Srrm2
Atxn2	Ddx28	**Hnrnpl**[a]	Npm3	Strap
Cdk9	Ddx56	**Igf2bp2**[a]	Polr2e	Zcchc24
Cdkn2aip	Ddx58	Ints6	Ppp1r12b	Znf281
Cpsf2	Eif4e2	Lsg1	Rbm26	Zrsr1;Zrsr2

[a] Bold gene names denote genes found in both *Hilnc*-bound top 30 candidates (left) and *Ucp1* mRNA-bound top 30 candidates (right) lists (Table S1).

Table S3.

Comprehensive Mass Spectrometry Data for *Hilnc*-bound and *Ucp1* mRNA-bound Proteins, Related to Figure 4.

**Gene names**	**Intensity Ctrl**	**Intensity Hilnc**	**Relative Intensity**
Hnrnpk	16197000000	1.8157E+11	11.21010064
Ptbp1	9077050000	1.558E+11	17.16416677
Hdlbp	16881000000	86913000000	5.148569398
Pcbp2	2604600000	59450000000	22.82500192
Igf2bp2	4572100000	45420000000	9.934165919
Hnrnpl	20430150000	42939000000	2.101746683
Matr3	1395990000	27285000000	19.54526895
Khsrp	4218400000	19367000000	4.591077186
U2af2	5808300000	15477000000	2.664635091
Khdrbs1	4011595000	13890000000	3.462463185
Rtcb	5794550000	13287000000	2.293016714
Fubp3	1877565000	12707000000	6.767808305
Sf1	3405505000	11515000000	3.38129
Pcbp1	590705000	11363000000	19.23633624
Rrp36	0	10880000000	#DIV/0!
Hnrnpd	2856140000	9267400000	3.2447289
U2af1	3692000000	9245300000	2.504144095
Ptbp3	325315000	8493200000	26.10761877

Rpl22	256450000	7964800000	31.05790602
Zc3h4	348285000	6795000000	19.50988415
Pabpn1;Gm20521	688040000	6673600000	9.699436079
Fubp1	2121795000	6607500000	3.114108573
Hnrnpll	1289550000	6164500000	4.780349734
Plec	807890000	5443700000	6.7381698
Puf60	1606000000	5387200000	3.354420922
Ago2	817290000	5377100000	6.57918242
Ptbp2	0	5327600000	#DIV/0!
Raver1	629285000	4386900000	6.971245143
Csde1	976140000	4364600000	4.471284857
Mbnl1	1044695000	4155200000	3.977428819
Nudt21	1771215000	4098300000	2.313835418
Fip1l1	594285000	3940100000	6.62998393
Ninl	0	3437900000	#DIV/0!
Pabpc4	796600000	3348700000	4.203740899
Gm17669;Rpl29;Gm3550	918800000	2898200000	3.154331737
Rbms2	1080625000	2714800000	2.512249855
Xrn2	598540000	2456700000	4.104487586
Vapa	392475000	2262900000	5.765717562
Pcbp3	52080000	2236400000	42.94162826
Celf2	25150500	2133000000	84.80944713
Wdr33	66355000	2052600000	30.93361465
Cstf3	264969500	2048600000	7.731455885
Cpsf1	52791500	2043700000	38.71267155
Wdr82	69377000	2021100000	29.13213313
Srrm2	368760000	1826200000	4.952272481
Qki	64970000	1821800000	28.04063414
Srpr	59877500	1819800000	30.39205044
Patl1	43765500	1771900000	40.48622774
Strap	354614500	1633300000	4.605846631
Cpsf7	606100000	1560400000	2.574492658
Ago1	208553500	1506100000	7.221648162
Srsf11	623295000	1453400000	2.331801154
Ccar1	495010000	1379600000	2.787014404
Xrn1	53875000	1214700000	22.54663573
Pkn3	98095000	1211600000	12.35129211
Gm10094;Sap18	319305000	1203000000	3.767557664
Celf1	0	1075700000	#DIV/0!
Cpsf2	57985000	1073300000	18.50995947
Hsd17b12	189820000	1049800000	5.530502581
Tnrc6b	76010000	1002900000	13.19431654

Ppp1r10	269239500	990550000	3.679066407
Hspa9	327955000	983160000	2.997850315
Eif4g2	458965000	980750000	2.136873182
Tecr	350605000	973140000	2.775602173
Zc3h13	294755000	963860000	3.270037828
Hadh	271635000	929540000	3.422018517
S100a6	339005000	920180000	2.714355245
Nup35	327150000	911900000	2.787406389
Lsm4	47107500	874810000	18.57050364
Lsm6	132555000	862930000	6.509976991
Cstf2;Cstf2t	13709000	733520000	53.50645561
Rpl6	74197000	726460000	9.790961899
Hadha	135505500	698260000	5.15300117
Atxn2	62793000	697940000	11.11493319
Fn1	130805000	674770000	5.158594855
Ppil1	155090000	673640000	4.343542459
Tial1	141150000	665360000	4.713850514
Rpl7	222771000	664400000	2.982434877
Srek1	85244500	649840000	7.623248421
Cpsf6	117190000	630490000	5.380066559
Rbm27	148079000	613240000	4.141302953
Pcbp2	31117000	597120000	19.18951056
Ppfibp1	68326500	561350000	8.215699619
Rpl7l1	55287000	556020000	10.05697542
Cstf1	34862000	555150000	15.92421548
March5	31094500	528890000	17.00911737
Pnn	83015500	526320000	6.340020839
Pcf11	15734500	524130000	33.31087737
Rbm26	17961000	522760000	29.10528367
Wtap	98326000	519620000	5.284665297
Gm20425;Srprb	216744500	482410000	2.225708149
Ylpm1	24405500	478630000	19.61156297
Eps8	105959000	476310000	4.495229287
Arhgef2	82005000	453650000	5.531979757
Lsm7	3479450	422970000	121.5623159
Lsm2	37610000	408460000	10.86040947
Atp5i;Atp5k	0	399310000	#DIV/0!
Slc25a11	113985000	394320000	3.459402553
Ctsb	185683500	393190000	2.117527944
Eif4a1;Eif4a2	151447500	379470000	2.50562076
Rpsa	137855000	375880000	2.726633056
Ddx56	131245000	375670000	2.862356661
Hsd17b4	98772500	367790000	3.723607279

Lsm3	10981000	364790000	33.22010746
Cdkn2aip	41881500	348660000	8.324916729
Vat1	31613500	326010000	10.31236655
Polrmt	104438000	324890000	3.110840882
Mpc2	112508500	323780000	2.877827009
Enah	25749000	308480000	11.98027108
Ankrd17	35567500	303250000	8.526042033
1700009N14Rik;Rasl2-9;Ran	53380000	290670000	5.445297864
Rpl28	32243500	289660000	8.983516057
Edf1	86350000	278860000	3.229415171
Polr2e	27736500	267110000	9.630270582
Rpl39	50140000	254630000	5.078380535
Vapb	0	254060000	#DIV/0!
Lsm1	0	234480000	#DIV/0!
Ncoa5	0	229060000	#DIV/0!
H2afy2	0	227320000	#DIV/0!
Nup88	51683500	227140000	4.394826202
Ints6	6858000	224060000	32.67133275
Fam98c	9162500	221080000	24.12878581
Gemin2	16648000	217430000	13.06042768
Myl6;Myl6b	44093000	213070000	4.832286304
Zswim8	0	208080000	#DIV/0!
Ddx28	0	206710000	#DIV/0!
Macf1	36278000	205870000	5.674789128
Aifm1	61837500	204310000	3.303982211
Hadhb	0	202150000	#DIV/0!
Gm7221;2610305D13Rik;Znf12;Zbtb41	0	201140000	#DIV/0!
Maged1	0	200840000	#DIV/0!
Kpnb1	41302000	199980000	4.841896276
Wbp11	68825000	198880000	2.889647657
Pdk1	0	198680000	#DIV/0!
Ube2i	0	198370000	#DIV/0!
Htra2	0	193180000	#DIV/0!
Tia1	21298000	188950000	8.871725045
Dnajc2	49980000	186890000	3.739295718
Celf2	0	179910000	#DIV/0!
Nop10	44388500	176090000	3.967018485
Mycbp	43758500	176020000	4.022532765
Polr2b	59392500	170550000	2.871574694
Larp1	9165500	168170000	18.3481534
Znfx1	0	167360000	#DIV/0!

Dido1	7346500	164980000	22.45695229
Ascc3	11652500	162970000	13.98583995
Hdgf	11646500	162170000	13.92435496
Angel2	0	161160000	#DIV/0!
Srbd1	17375500	161010000	9.266495928
Dpm1	39370500	158720000	4.031444864
Thoc2;BC005561	60023500	157210000	2.619140837
Prdx4	49102500	150510000	3.065220712
Dnaja2	0	148320000	#DIV/0!
Zcchc24	46525000	147700000	3.174637292
Tspo	17642000	145850000	8.267203265
Rbm39	0	145050000	#DIV/0!
Zrsr1;Zrsr2	0	144530000	#DIV/0!
Eif3l	0	138100000	#DIV/0!
Vdac2	31021500	137820000	4.442725207
Znf281	14814000	136710000	9.228432564
Chchd1	23195000	134050000	5.779262772
Atp5l	0	132240000	#DIV/0!
Ttc37	0	130940000	#DIV/0!
Fam195b	0	124010000	#DIV/0!
Rnaseh1	0	119840000	#DIV/0!
Gltscr2	0	115580000	#DIV/0!
Zcrb1	17226000	115120000	6.682921166
Tfip11	15194500	115100000	7.575109415
Col6a3	21793000	113520000	5.209012068
Sh3pxd2b	45209500	113050000	2.50058063
Kdelc1	21496000	109340000	5.086527726
Mark1	0	109260000	#DIV/0!
Rfc3	16191000	109010000	6.732752764
Nr2f2;Nr2f1	26510050	107850000	4.068268449
Rab8a;Rab1;Rab15;Rab10;Rab1b;Rab13;Rab1A;Rab8b	11594500	106220000	9.161240243
Prps1l1	0	104940000	#DIV/0!
Ruvbl2	10351000	104260000	10.07245677
Cbr4	22757000	102150000	4.488728743
Mrpl1	0	101530000	#DIV/0!
Pin4	6883500	101130000	14.69165396
Yme1l1	0	98176000	#DIV/0!
Gatad2a	0	96801000	#DIV/0!
Cdk16;Cdk17	0	96719000	#DIV/0!
Ssr1	0	96111000	#DIV/0!
Ddx58	11571000	93430000	8.074496586

Mrpl9	0	92945000	#DIV/0!
Mbd3	0	91638000	#DIV/0!
Camk2d;Camk2a;Camk2b;Camk2g	0	91141000	#DIV/0!
Tnrc6c	0	89206000	#DIV/0!
Usmg5	15083500	87676000	5.812709252
Eif4enif1	33811500	86441000	2.556556201
Serf2;Serf1	29671000	86338000	2.909844629
Ccar2	0	82816000	#DIV/0!
Zc3h11a	0	82772000	#DIV/0!
Rfc5	0	78896000	#DIV/0!
Numbl	0	78526000	#DIV/0!
Rbm45	0	75354000	#DIV/0!
Col6a1	22219500	74248000	3.341569342
1810022K09Rik	24294500	74191000	3.053818766
Cpsf4	0	69938000	#DIV/0!
Dnajb4	0	67983000	#DIV/0!
Pcbp4	0	67569000	#DIV/0!
Larp4b	14617000	66765000	4.567626736
Samhd1	0	64874000	#DIV/0!
Nxt1	18619500	64222000	3.449179624
Pdcd7	16268500	63974000	3.93238467
Gramd4	0	63958000	#DIV/0!
Strbp	0	60510000	#DIV/0!
Nup107	0	59260000	#DIV/0!
Mbnl1	0	59179000	#DIV/0!
P4ha1	0	59175000	#DIV/0!
Ascc1	15616500	58909000	3.772228092
2010002M12Rik;Gm14446;Ifit1	0	58182000	#DIV/0!
Cbx6	0	58046000	#DIV/0!
Elmsan1	0	57604000	#DIV/0!
Inip	0	55623000	#DIV/0!
Dis3	13583000	54663000	4.024368696
Cggbp1	20694000	54420000	2.629747753
Rnaseh2b	0	54186000	#DIV/0!
Safb2	0	52617000	#DIV/0!
Brd4	2969600	50049000	16.85378502
Cdc40	0	49161000	#DIV/0!
Ppp1r12b	0	48912000	#DIV/0!
Wdr61	0	48480000	#DIV/0!
Arpc5l	0	48134000	#DIV/0!
Reep3	0	45743000	#DIV/0!

Ppp1cc	0	45694000	#DIV/0!
Vti1b	0	45032000	#DIV/0!
Ldha	0	40971000	#DIV/0!
Hmga2	0	40658000	#DIV/0!
Samd1	0	40385000	#DIV/0!
Slc30a9	0	39440000	#DIV/0!
Ppp2r1a;Ppp2r1b	13241500	39191000	2.959710003
Srsf6;Srsf4	0	39001000	#DIV/0!
Znf830	0	38947000	#DIV/0!
Lsg1	16865500	38442000	2.279327621
Gpatch1	0	37240000	#DIV/0!
Ddx10	4683100	36274000	7.745723986
Camsap2	12015500	35912000	2.988806125
Mmaa	0	35125000	#DIV/0!
Eif4e2	16887000	34821000	2.062000355
Slc2a1	0	34308000	#DIV/0!
Mocs1	0	34268000	#DIV/0!
Kpna4	0	34135000	#DIV/0!
P4hb	0	33951000	#DIV/0!
Cdk9	0	32959000	#DIV/0!
Morf4l2	0	32926000	#DIV/0!
Alg2	0	32312000	#DIV/0!
Clasp1	0	32284000	#DIV/0!
Skp1	0	32076000	#DIV/0!
Ints3	10290500	30963000	3.008891696
Phf6	0	30529000	#DIV/0!
Nupl2	0	29968000	#DIV/0!
Utp18	14192000	29936000	2.109357384
Lsm8	0	29293000	#DIV/0!
Rai14	0	29113000	#DIV/0!
Txlna	0	28820000	#DIV/0!
Cpne8	10152000	28672000	2.82427108
Col6a2	0	28166000	#DIV/0!
Ckap2	0	27663000	#DIV/0!
Ccdc47	0	27108000	#DIV/0!
Mtch2	0	27006000	#DIV/0!
Mndal	0	25516000	#DIV/0!
Bud13	0	25228000	#DIV/0!
Flna;Flnb;Flnc	0	24799000	#DIV/0!
S100a11	5904000	24549000	4.158028455
Vdac1	0	24488000	#DIV/0!
Obsl1	0	23763000	#DIV/0!
Calu	0	23762000	#DIV/0!

Ywhab	0	23700000	#DIV/0!
Fhl3	0	22910000	#DIV/0!
Bysl	10387000	22676000	2.183113507
Mcm3ap	0	22321000	#DIV/0!
Dnajb12	0	21526000	#DIV/0!
Kif5a;Kif5b;Kif5c	0	20891000	#DIV/0!
Epb41l3	0	19071000	#DIV/0!
Heatr3	0	17325000	#DIV/0!
Cep55	0	16474000	#DIV/0!
Anxa2	0	15648000	#DIV/0!
Npm3	0	15538000	#DIV/0!
Gba	0	15252000	#DIV/0!
Arid5b	0	14843000	#DIV/0!
Ccdc80	0	11409000	#DIV/0!
Khnyn	0	8194900	#DIV/0!
Gtf2h1	0	7734900	#DIV/0!

**Gene names**	**Intensity Ctrl**	**Intensity Ucp1-mRNA**	**Relative Intensity**
Actbl2	91307000	39070000000	427.8970944
Eif2ak2	881250000	17328000000	19.66297872
Ybx1	3906200000	11582000000	2.965029952
Igf2bp3	5180700000	10379000000	2.003397224
Hnrnpl	1603400000	9367400000	5.842210303
Pccb	3643400000	8233100000	2.259729923
Igf2bp2	2917000000	6715800000	2.30229688
Luc7l2	3023800000	6123100000	2.024968583
Ybx3	1051600000	4545400000	4.322365919
Syncrip	1932800000	4532600000	2.345095199
Hnrnpa0	1299800000	2904400000	2.234497615
Hmgb1	803970000	1946300000	2.420861475
Hnrnpr	342250000	1892200000	5.528707085
Ptn	119640000	1804400000	15.0819124
Zfr	340240000	1743500000	5.124324007
Lsg1	678870000	1628200000	2.398397337
Arpc5	386410000	1448600000	3.748867783
Atxn2l	521910000	1250700000	2.396390182
Hnrnpf	554290000	1239500000	2.23619405
2010002M12Rik;Gm14446	206510000	1162600000	5.629751586
Helz2	147580000	1126500000	7.633148123
Syne3	0	1118800000	#DIV/0!
Snrnp40	492750000	1028900000	2.088077118

Zfp948;Zfp708;Zfp273;3110052M02Rik;Zfp85;Zfp729b;Gm15446;Gm10226;Zfp947;Zfp677;Zfp108;Zfp160;Gm4944;Zfp235;Zfp493;Zkscan7;Zfp40;2610021A01Rik;Zfp658;Zfp758;2210404O09Rik;Zfp773;B020011L13Rik;Zfp78;Zfp61;Znf271;Znf431;Zfp111;Zfp72;Znf235;Zfp62;Znf664;Znf513;Znf287;Zfp791;Znf112;Zfp114;Zfp51;Zscan21;Zfp58;Zkscan5;Zfp28;Znf24;Zfp53;Znf322;Znf18;Znf354c;Znf319;Zfp729a;Zfp738;Zfp790;Rslcan18	0	1011400000	#DIV/0!
Srsf9	379040000	987750000	2.605925496
Rplp1	119180000	967640000	8.119147508
Ddx56	409330000	938530000	2.292844404
Nufip2	416590000	841930000	2.021003865
Cacna1a	0	839170000	#DIV/0!
Cpsf2	392880000	823060000	2.094939931
Ccnk	289380000	817460000	2.824866957
Rbm15	115810000	801560000	6.921336672
Kctd10	352130000	779180000	2.212762332
Ran;1700009N14Rik	306920000	768500000	2.503909814
Ddx51	368340000	752390000	2.042650812
Hbs1l	0	738060000	#DIV/0!
Ago2	187860000	712440000	3.792398595
Rbms1;Rbms3	302000000	710230000	2.351754967
Capn6	348470000	708230000	2.032398772
Btf3l4	69576000	704880000	10.13107968
Lcp1	333310000	694990000	2.085115958
Csde1	185740000	678900000	3.655109293
Tusc3	143390000	611290000	4.263128531
Tpi1	260290000	600640000	2.307580007
Nsun5	224590000	579970000	2.58235006
Srsf7	131790000	552750000	4.194172547
Hnrnpul1	0	547580000	#DIV/0!
Snrnp70	232660000	543560000	2.336284707
Ppan	183910000	543150000	2.953346746
Ppp1r9b	159250000	523720000	3.28866562

Ak1	184780000	520960000	2.819352744
Znf512	123650000	505210000	4.085806712
Pura	231000000	505110000	2.186623377
Hnrnpul2	35183000	477610000	13.57502203
Znf281	63843000	458580000	7.182933133
Sfpq	109120000	441470000	4.045729472
Gnl2	157940000	438620000	2.777130556
Eri1	177720000	435780000	2.452059419
Enthd1	0	409590000	#DIV/0!
Larp4	172750000	405650000	2.348191027
Epb41l5	48871000	402940000	8.244971455
Gemin5	101720000	399540000	3.927841133
Atxn2	190060000	398880000	2.098705672
Raly	142770000	386560000	2.707571619
Eif4g1	55908000	380770000	6.810653216
Knop1	0	373110000	#DIV/0!
Ppp2ca;Ppp2cb	0	353660000	#DIV/0!
Srrm2	123750000	344640000	2.784969697
Ccdc137	97804000	334910000	3.424297575
Hnrnph2	48575000	321930000	6.627483273
H2afx	0	311770000	#DIV/0!
Phf5a	0	303930000	#DIV/0!
Sfswap	0	303350000	#DIV/0!
Ddx50	51655000	301360000	5.834091569
Afg3l1	75501000	296100000	3.92180236
Srsf5	0	286970000	#DIV/0!
Dhx37	100310000	286060000	2.851759545
Glg1	76060000	284090000	3.73507757
Prps2	45648000	270710000	5.930380301
Strap	0	265730000	#DIV/0!
Plcd3	74437000	261250000	3.509679326
Gpatch4	83100000	257030000	3.093020457
Atp6v0d1	123130000	252460000	2.050353285
Tarbp2	74535000	251680000	3.376668679
Dnmt1	80970000	251000000	3.099913548
Uggt2	102380000	241360000	2.357491698
Fam32a	42440000	234480000	5.524976437
Snrpe	74883000	228580000	3.052495226
Chchd3	30995000	228020000	7.356670431
Ass1	0	221500000	#DIV/0!
Rnps1	41403000	214690000	5.185373041
Sltm	40110000	213130000	5.313637497
Pxdn	0	212340000	#DIV/0!

Cdc37	57227000	211840000	3.701749174
Plekhf1	0	202230000	#DIV/0!
Csnk1d;Csnk1e	0	200950000	#DIV/0!
Syde1	75569000	193340000	2.55845651
Poldip3	42937000	192930000	4.493327433
Pip5k1a	0	188430000	#DIV/0!
Fam98b	93553000	187800000	2.007418255
Tagln2	65944000	187130000	2.837710785
Rbbp6	66147000	184880000	2.794986923
Golga3	46877000	184150000	3.928365723
Sf3b4	0	182950000	#DIV/0!
Rhot1	0	180520000	#DIV/0!
Zcchc24	0	179870000	#DIV/0!
Zc3h15	57105000	179470000	3.14280711
Dpy30	0	174860000	#DIV/0!
Tcirg1	79957000	167030000	2.088997836
Vdac3	0	166360000	#DIV/0!
Eif3i	46019000	165990000	3.606988418
Rcn3	72903000	162030000	2.222542282
Skiv2l2	0	161130000	#DIV/0!
Cand2	0	160750000	#DIV/0!
Ddx58	47316000	158310000	3.345802688
Dnajc21	0	157180000	#DIV/0!
Fam193a	59433000	153320000	2.579711608
Prpf38a	0	151930000	#DIV/0!
Cdk9	0	151930000	#DIV/0!
Atxn10	0	146800000	#DIV/0!
Ankfy1	61796000	146380000	2.368761732
Ercc6l2	0	144760000	#DIV/0!
Rad21l1	0	141680000	#DIV/0!
Mark3	66005000	141210000	2.13938338
Yes1;Src;Fyn	68916000	140420000	2.037552963
Fubp3	53806000	139840000	2.598966658
Cdc42	39327000	139550000	3.548452717
Tdrd3	49881000	139540000	2.79745795
Pak1ip1	0	138670000	#DIV/0!
Prkar2b;Prkar2a	25482000	138210000	5.423828585
Hmmr	42002000	135290000	3.221037093
Gsdmc3;Gsdmc4;Gsdmc2	0	135260000	#DIV/0!
Irgm1	59041000	134080000	2.270964245
Bclaf1	56171000	129340000	2.302611668
Ptpn14	27552000	128460000	4.662456446

Naa38	0	128030000	#DIV/0!
Trip4	33406000	127630000	3.820571155
Slirp	0	126230000	#DIV/0!
Sdhb	51161000	126180000	2.466331776
Rac1	0	122400000	#DIV/0!
Ipo5	0	122100000	#DIV/0!
Sbf2	0	122070000	#DIV/0!
Utp20	47675000	121810000	2.555007866
Rasa4	52686000	120230000	2.282010401
Slc3a2	57311000	120210000	2.097503097
Dhx58	24219000	115650000	4.775176514
Memo1	51723000	114660000	2.21680877
Paxbp1	48606000	114090000	2.347241081
Dvl3	32209000	113230000	3.515477041
Trap1	44875000	112900000	2.515877437
Snap47	42556000	112480000	2.643105555
Mphosph10	0	110390000	#DIV/0!
Map4	0	109700000	#DIV/0!
Aatf	0	109470000	#DIV/0!
Vrk2	0	107950000	#DIV/0!
Ddx49	0	105740000	#DIV/0!
Zbtb7a	26876000	104310000	3.88115791
Ints6	49419000	104240000	2.109310184
Uncharacterized protein C17orf62 homolog	50188000	103460000	2.061448952
Cd63	49306000	102440000	2.07763761
Tex10	0	102410000	#DIV/0!
Psat1	43601000	100710000	2.309809408
Znf148	49331000	100060000	2.028339178
Imp4	0	95356000	#DIV/0!
Brms1l	0	94854000	#DIV/0!
Faf2	0	94598000	#DIV/0!
Eif4e2	27989000	94526000	3.37725535
Dpysl2	0	92760000	#DIV/0!
Npm3	0	92380000	#DIV/0!
Mfsd10	0	90477000	#DIV/0!
Prkca	0	89490000	#DIV/0!
UPF0428 protein CXorf56 homolog	0	89154000	#DIV/0!
Msto1	41592000	88308000	2.123196769
Spz1	0	87526000	#DIV/0!
Zrsr1;Zrsr2	0	85841000	#DIV/0!
Gkap1	0	84741000	#DIV/0!

S100a13	0	79024000	#DIV/0!
Znhit3	0	78784000	#DIV/0!
Gtpbp1	33043000	78485000	2.375238326
Ddx28	28007000	78295000	2.795551112
Dcaf13	0	76943000	#DIV/0!
Eif3m	0	76860000	#DIV/0!
Tes	34756000	74644000	2.147657958
Rbm26	0	72572000	#DIV/0!
Onecut2	0	71689000	#DIV/0!
Polr2e	23101000	71585000	3.098783602
Pfkp	0	71062000	#DIV/0!
Cebpb	0	69704000	#DIV/0!
Ppfia1	0	69548000	#DIV/0!
Xpo5	0	69071000	#DIV/0!
Son	0	67210000	#DIV/0!
Baz1a	0	67112000	#DIV/0!
Wdr5	0	66858000	#DIV/0!
Clk3	0	65971000	#DIV/0!
Farsb	0	65078000	#DIV/0!
Mettl16	0	63671000	#DIV/0!
Mrpl9	0	61961000	#DIV/0!
Acot9;Acot10	0	61891000	#DIV/0!
Ctsk	0	61580000	#DIV/0!
Fosl2	0	61556000	#DIV/0!
Cbr1	0	59650000	#DIV/0!
Bap18	0	59139000	#DIV/0!
Nrde2	0	58909000	#DIV/0!
Rbm8a	0	58844000	#DIV/0!
Dock2	0	58349000	#DIV/0!
Ltv1	0	58314000	#DIV/0!
Riok2	0	58168000	#DIV/0!
Chd1;Chd2	0	57277000	#DIV/0!
Raph1	0	56290000	#DIV/0!
Mtpn	0	55810000	#DIV/0!
Coro1b	0	54868000	#DIV/0!
Mthfd1	0	54449000	#DIV/0!
Kifc1;Kifc5b	0	53791000	#DIV/0!
Ccnt1	0	53770000	#DIV/0!
Samd4a	0	52299000	#DIV/0!
Gemin4	0	51604000	#DIV/0!
Dhx38	0	51345000	#DIV/0!
Efr3a	0	51231000	#DIV/0!
Capns1	0	51230000	#DIV/0!

Trmt1l	0	50963000	#DIV/0!
Mical2	0	50536000	#DIV/0!
Myo7a	0	50192000	#DIV/0!
Znf787	0	49446000	#DIV/0!
Gtpbp10	0	49106000	#DIV/0!
Herc6	0	49086000	#DIV/0!
Usp7	21685000	48892000	2.254646069
Ppp1r12b	0	46713000	#DIV/0!
Exosc7	0	44946000	#DIV/0!
Ythdc1	0	44804000	#DIV/0!
Tmed9	0	44118000	#DIV/0!
Loxl4	0	42194000	#DIV/0!
Gpatch8	0	42145000	#DIV/0!
Kif2c	0	41779000	#DIV/0!
Cd2bp2	0	41471000	#DIV/0!
Carhsp1	0	39590000	#DIV/0!
Ascc2	0	39575000	#DIV/0!
Ppp6r3	0	38661000	#DIV/0!
UPF0488 protein C8orf33 homolog	0	38396000	#DIV/0!
Ipo9	0	37453000	#DIV/0!
Aurkb	0	37441000	#DIV/0!
Ddx20	0	37355000	#DIV/0!
Acadvl	0	37151000	#DIV/0!
Junb	0	36284000	#DIV/0!
Kif14	0	35936000	#DIV/0!
Mrps7	0	33875000	#DIV/0!
Pdia6	0	31318000	#DIV/0!
Zfp655	13041000	30997000	2.376888275
Cacna2d1	0	30323000	#DIV/0!
Smu1	0	29603000	#DIV/0!
Calr	0	27313000	#DIV/0!
Mrps5	0	26836000	#DIV/0!
Dctn2	0	25589000	#DIV/0!
Noc3l	0	25017000	#DIV/0!
Uaca	0	24330000	#DIV/0!
Smarca2	0	23996000	#DIV/0!
Srek1ip1	0	23916000	#DIV/0!
Dst	0	23795000	#DIV/0!
Thoc6	0	23280000	#DIV/0!
Pawr	0	22515000	#DIV/0!
Lig1	0	22224000	#DIV/0!
B4galt6	0	21345000	#DIV/0!

Srsf4	0	18644000	#DIV/0!
Bhlhb9	0	16555000	#DIV/0!
Cdkn2aip	0	15357000	#DIV/0!

Table S4.
List of the primers used in the study.

Primer name	sequence
mActin-realf	GCAGGAGTACGATGAGTCCG
mActin-realr	ACGCAGCTCAGTAACAGTCC
m18s-realf	CCGCCGCCATGTCTCTAGT
m18s-realr	CTTTCCTCAACACCACATGAGC
Hilnc-realf	CTGGACCTGTCAATCACCTCG
Hilnc-realr	GGGACTTCTCAGGGAGGTACA
mUcp1-realf	ACTGCCACACCTCCAGTCATT
mUcp1-realr	CTTTGCCTCACTCAGGATTGG
mPparg-realf	TCGCTGATGCACTGCCTATG
mPparg-realr	GAGAGGTCCACAGAGCTGATT
mFabp1-realf	ATGAACTTCTCCGGCAAGTACC
mFabp1-realr	CTGACACCCCCTTGATGTCC
mFgf21-realf	AAAGCCTCTAGGTTTCTTTGCCA
mFgf21-realr	CCTCAGGATCAAAGTGAGGCG
mt-Nd3-realf	GTTGCATTCTGACTCCCCCA
mt-Nd3-realr	GGTAGACGTGCAGAGCTTGT
mt-Atp8-realf	AACATTCCCACTGGCACCTT
mt-Atp8-realr	TGTTGGGGTAATGAATGAGGCA
mt-Co3-realf	GGCCACCACACTCCTATTGT
mt-Co3-realr	CGCTCAGAAGAATCCTGCAAAG
mt-Nd4l-realf	ACTCCAACTCCATAAGCTCCA
mt-Nd4l-realr	TAGTCCTACAGCTGCTTCGC
Opa1-realf	GCTCAGAAGACCTTGCCAGT
Opa1-realr	CCTAACAAGAGAAGGGCCTCA
Aifm1-realf	CGAGGAGTGATCGCCGAAAT
Aifm1-realr	GAACACGCCATTGCTGGAAC
Cox4i1-realf	CCCTGATTCCCGCGATGCTT
Cox4i1-realr	GCTCTCTTGCCAATCAGGCT
mPrdm16-real-f	CAGCACGGTGAAGCCATTC
mPrdm16-real-r	GCGTGCATCCGCTTGTG
mPGC1a-real-f	AGCCGTGACCACTGACAACGA
mPGC1a-real-r	GCTGCATGGTTCTGAGTGCT
mCidea-real-f	ATCACAACTGGCCTGGTTACG
mCidea-real-r	TACTACCCGGTGTCCATTTCT
mC/EBPa-realf	AATGGCAGTGTGCACGTCTA

mC/EBPa-realr	CCCCAGCCGTTAGTGAAGAG
hActin-realf	CATGTACGTTGCTATCCAGGC
hActin-realr	CTCCTTAATGTCACGCACGAT
hHilnc-real5f	ATCCAACAATAATAACTAAAGAGGCAAGG
hHilnc-real5r	AACTTTAGACAAACTGTGAGTTCCAC
hUCP1-real f1	AGGTCCAAGGTGAATGCCC
hUCP1-real r1	TTACCACAGCGGTGATTGTTC
T7-hHilncfl-F	TAATACGACTCACTATAGGGTTCTTGTGAT TAGCAATC
hHilncfl-F	TTCTTGTGATTAGCAATCAAGGACAG
hHilncfl-R	TGGTTTTAAAAATTTAACTTTAGACTATGT ATGCATATGC
T7-Hilnc 1-150 For	AGTCAGCTCGTAATACGACTCACTATAGG GAACTCACTGCGTACTTTG
Hilnc1-150 Rev	CAGGAATGTGGCGCCAGTTTAG
T7-Hilnc 150-300 For	AGTCAGCTCGTAATACGACTCACTATAGG GGCTGGAACTCTGTCACAGTC
Hilnc 150-300 Rev	ACTCAGTGTTTACGAGGCTTAAG
T7-Hilnc 300-450 For	AGTCAGCTCGTAATACGACTCACTATAGG GTGAGTCCAGCTGGACCTGTC
Hilnc 300-450 Rev	GGACTGAGGGAGGGACTTCTC
T7-Hilnc 450-600 For	AGTCAGCTCGTAATACGACTCACTATAGG GCAGCTGTTTGCAGATCTTTC
Hilnc 450-600 Rev	GGTTAAAGAAAGAGAGTGTAC
T7-Hilnc 600-783 For	AGTCAGCTCGTAATACGACTCACTATAGG GCCCCTCCAAGCCCCAGTTAC
Hilnc Rev	GGAAGCTTGTGGACTCTTTTATTG
T7-hUCP1-FL-For	AGTCAGCTCGTAATACGACTCACTATAGG AGAGGGTCCTGCTGGCGCGAG
hUCP1-5'UTR-Rev	CTTCACTCAGAGACTGGAGATG
T7-hUCP1-CDS-For	AGTCAGCTCGTAATACGACTCACTATAGG ATGGGGGGCCTGACAGCCTC
hUCP1-CDS-Rev	TTATGTGGCACAGTCCATAG
T7-hUCP1-3'UTR-For	AGTCAGCTCGTAATACGACTCACTATAGG TCAGCTTCAAGAAAATGATGTAACATACC AG
hUCP1-FL-Rev	TTTAAAAGGTATTAGCAATAC
T7-mUCP1 FL For	AGTCAGCTCGTAATACGACTCACTATAGG AAGTGCCGGGCAATCTG
mUCP1 5'UTR Rev	CCTGGCTTGGAGGGCAGAGAG
T7-mUCP1 CDS For	AGTCAGCTCGTAATACGACTCACTATAGG ATGGTGAACCCGACAACTTCCG
mUCP1 CDS Rev	TTATGTGGTACAATCCACTG

T7-mUCP1 3'UTR For	AGTCAGCTCGTAATACGACTCACTATAGG GCAACTTGGAGGAAGAGATAC
mUCP1 FL Rev	AGATGGAATTAGCAATACTTTATTG
Ucp1-Cre-F	CGATGCAACGAGTGATGAGG
Ucp1-Cre-R	CGCATAACCAGTGAAACAGC
Hilnc-flox-F1	TGAGCACCAATCATAGGCTGC
Hilnc-flox-R1	CCAACTGACCTTGGGCAAGAACAT
Hilnc-flox-F2	CCCGAAGCATCTGAATTTAGAGC
Hilnc-flox-R2	CTGGTCATTCTGAGACATAGCCTG
common cre f	ATCCGAAAAGAAAACGTTGA
common cre r	ATCCAGGTTACGGATATAGT
